# Critical nematic correlations throughout the superconducting doping range in Bi_2−*z*_Pb_*z*_Sr_2−*y*_La_*y*_CuO_6+*x*_

**DOI:** 10.1038/s41467-023-38249-3

**Published:** 2023-05-05

**Authors:** Can-Li Song, Elizabeth J. Main, Forrest Simmons, Shuo Liu, Benjamin Phillabaum, Karin A. Dahmen, Eric W. Hudson, Jennifer E. Hoffman, Erica W. Carlson

**Affiliations:** 1 Department of Physics, Harvard University, Cambridge, MA 02138 USA; 2 Department of Physics and Astronomy, Purdue University, West Lafayette, IN 47907 USA; 3 Purdue Quantum Science and Engineering Institute, West Lafayette, IN 47907 USA; 4 Department of Physics, University of Illinois, Urbana-Champaign, IL 61801 USA; 5 Department of Physics, The Pennsylvania State University, University Park, PA 16802 USA

**Keywords:** Superconducting properties and materials, Phase transitions and critical phenomena

## Abstract

Charge modulations have been widely observed in cuprates, suggesting their centrality for understanding the high-*T*_*c*_ superconductivity in these materials. However, the dimensionality of these modulations remains controversial, including whether their wavevector is unidirectional or bidirectional, and also whether they extend seamlessly from the surface of the material into the bulk. Material disorder presents severe challenges to understanding the charge modulations through bulk scattering techniques. We use a local technique, scanning tunneling microscopy, to image the static charge modulations on Bi_2−*z*_Pb_*z*_Sr_2−*y*_La_*y*_CuO_6+*x*_. The ratio of the phase correlation length *ξ*_CDW_ to the orientation correlation length *ξ*_orient_ points to unidirectional charge modulations. By computing new critical exponents at free surfaces including that of the pair connectivity correlation function, we show that these locally 1D charge modulations are actually a bulk effect resulting from classical 3D criticality of the random field Ising model throughout the entire superconducting doping range.

## Abstract (web summary)

Charge order has been widely observed in cuprate superconductors. Here, the authors conduct scanning tunneling microscopy measurements on (BiPb)2(SrLa)2CuO6+x and find that the charge order fills the bulk and is predominantly unidirectional with classical, rather than quantum, critical fluctuations.

## Introduction

Charge order (CO), long seen on the surface of Bi2212^1–3^, Bi2201^4^, and Na-CCOC^3,5^, has recently been demonstrated in the bulk of many superconducting cuprates by NMR and scattering techniques^6–18^. Its apparent universality prioritizes its microscopic understanding and the question of its relationship to superconductivity (SC). However, severe material disorder presents both a challenge and an opportunity^19^. The challenge is that material disorder disrupts long-range order and limits macroscopic experimental probes to reporting spatially averaged properties. In particular, while numerous theories rest upon the 1D (stripe) or 2D (checker) nature of the CO, bulk probes may collect signal from multiple domains, obscuring the underlying dimensionality within a single domain.

The opportunity is for local probes to employ disorder as a knob that spatially varies parameters such as doping and strain within a single sample, to test and quantify the relationship of CO to SC. However, this strategy rests on the premise that what is seen on the surface is not merely a surface effect, but is reflective of the bulk of the sample. In Bi2201, while CO has been observed in the bulk (via, e.g., resonant X-ray scattering^9^) with the same average wavevector as on the surface, it has not yet been demonstrated that they are locally the same phenomenon. That work also left open the question of whether the CO is locally unidirectional (“stripe-like”) or bidirectional (a “checkerboard”). Here, we combine a local probe, scanning tunneling microscopy (STM), with a theoretical framework known as cluster analysis^19^, appropriate near a critical point, in order to test whether the surface CO is connected to the bulk CO. We find that the charge modulations in Bi2201 have significant stripe character. By computing new critical exponents at free surfaces including that of the pair connectivity correlation function, we moreover show that these charge modulations pervade the bulk of the sample, and that their spatial correlations are critical throughout the doping range of superconductivity.

**Fig. 1:**
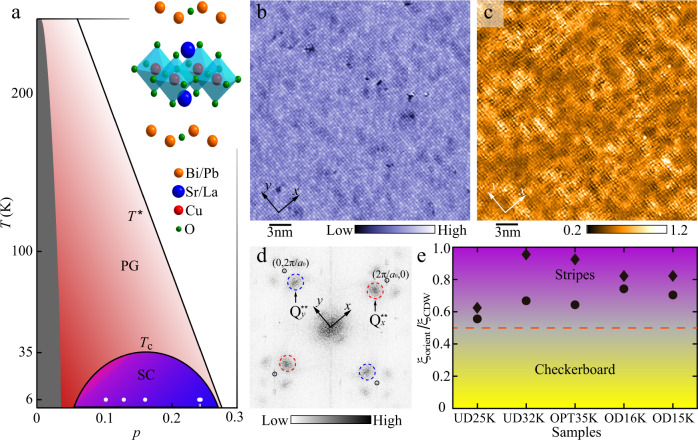
Phase diagram and stripe order. **a** Schematic temperature (*T*) versus chemical-doping (*p*) phase diagram of Bi2201, displaying both superconducting (SC) and pseudogap (PG) phases. Inset shows the crystal structure of Bi2201. The five white dots and thin lines denote the five samples studied, namely UD25K, UD32K, OPT35K, OD16K, and OD15K (from left to right), where *p* has been inferred from the measured *T*_*c*_^50^. **b** Constant-current STM topography of UD32K sample acquired at *I* = 400 pA and *V*_*s*_ = − 200 mV, over a 30 nm × 30 nm field of view. The arrows corresponds to the two orthogonal Cu-O bond directions throughout this paper. **c** A typical tunneling asymmetry *R*-map taken at 100 mV (i.e. *R*(r, 100 mV) = *I*(**r**, 100 mV)/*I*(**r**, − 100 mV)) in the same field of view shown in (**b**). A disordered charge modulation with a period of ~ 4*a*_0_ is evident in real space. **d** Fourier transform (FT) of *R*-map over the entire FOV in (**c**), with Bragg vectors (±1, 0)2*π*/*a*_0_ and (0, ±1)2*π*/*a*_0_ marked by black circles. The wavevectors ${Q}_{x}^{*\ast } \sim (3/4,0)2\pi /{a}_{0}$ and ${Q}_{y}^{*\ast } \sim (0,3/4)2\pi /{a}_{0}$ from the charge modulation are identified by dashed red and blue circles. **e** *ξ*_orient_/*ξ*_CDW_ extracted from two different definitions in Refs. ^32^ (circles) and ^33^ (diamonds). Ratios of *ξ*_orient_/*ξ*_CDW_ appear >0.5 for all samples, consistent with a striped nature of the charge order. The purple and yellow regions indicate the stripe and checkerboard phases, respectively.

We use STM to study the cuprate high-temperature superconductor Bi_2−*z*_Pb_*z*_Sr_2−*y*_La_*y*_CuO_6+*x*_ (Bi2201) at the dopings shown in Fig. 1a, from underdoped to overdoped superconducting samples, as well as an optimally doped sample with superconducting transition temperature *T*_*c*_ = 35 K, as a function of hole concentration *p*. Figure 1b shows a topographic image of slightly underdoped Bi2201 with transition temperature *T*_*c*_ = 32 K (UD32K), drift-corrected as described in Ref. ^20^. La and O doping supplies the holes, while Pb doping suppresses the structural supermodulation, leaving only the atomic corrugations with a periodicity of *a*_0_ = 3.8 Åbetween copper atoms in the Cu-O planes.

## Results

### Stripes vs. checkers

To identify the nature of the charge modulations, we focus on the *R*-map, where *R*(***r***, *V*) = *I*(***r***, *V*)/*I*(***r***, −*V*), and *I*(***r***, ±*V*) represents the STM tunneling current at ±*V* as a function of position ***r*** on the surface of the sample^3^. The *R*-map has the advantage that it cancels out certain unmeasurable quantities, such as the tunneling matrix element and tunnel barrier height. Figure 1c shows the *R*-map with *V* = 100 mV in the same field of view (FOV) as Fig. 1b. A local modulation with period near 4*a*_0_ is readily apparent, as confirmed by the two-dimensional Fourier transform (FT) of the *R*-map in Fig. 1d, showing peaks at ${Q}_{x}^{*\ast } \sim (\pm \!3/4,0)2\pi /{a}_{0}$ and ${Q}_{y}^{*\ast } \sim (0,\pm \!3/4)2\pi /{a}_{0}$. The Q^**^ peaks carry information about the same charge modulation as the peaks at ${Q}_{x}^{*} \sim (\!\pm \!1/4,0)2\pi /{a}_{0}$ and ${Q}_{y}^{*} \sim (0,\pm \!1/4)2\pi /{a}_{0}$^21, 22^, and because they are well-separated from the central broad FT peak, there is less measurement error associated with tracking Q^**^. We therefore focus on the Q^**^ peaks.

There has been experimental evidence in several families of cuprate superconductors for both stripe order (unidirectional CDW)^2,23–25^ and checkerboard order (bidirectional CDW)^1,26–28^. Recent experiments on YBCO show that the issue of dimensionality of the CO in cuprates can be quite complex: while the zero field CDW is 2D correlated, the field-induced CDW is both 3D correlated^29^ and unidirectional^30^. Recent experiments on LSCO point to a multi-faceted relationship between CO and SC: while short-range CO exists above the superconducting dome, the CO is unidirectional in the superconducting regime^31^. It is difficult to discern from direct observation which tendency (stripes or checkerboards) would dominate in a hypothetical zero disorder limit^32,33^, because the quenched disorder that is always present in real materials can favor the appearance of stripe correlations^33^. One metric for distinguishing whether the underlying electronic tendency favors stripes or checkerboards is to compare the correlation length of the periodic density modulations *ξ*_CDW_ with the correlation length of the orientation of the modulations *ξ*_orient_. Two different theoretical approaches^32,33^ predict that ${\xi }_{{{{{{{{\rm{orient}}}}}}}}}\, > \, \frac{1}{2}{\xi }_{{{{{{{{\rm{CDW}}}}}}}}}$ when the underlying tendency is toward stripes rather than checkerboard modulations.

In order to infer whether the charge modulations would tend toward stripes or checkerboards in Bi2201 in a hypothetical zero disorder limit, we construct the local Fourier components of the *R*-map at wavevector **q,**

1 $$A({{{{{{{\bf{q}}}}}}}},{{{{{{{\bf{r}}}}}}}})=\frac{1}{2{\pi }^{2}{L}^{2}}\int\,R({{{{{{{\bf{r}}}}}}}}){e}^{i{{{{{{{\bf{q}}}}}}}}\cdot {{{{{{{{\bf{r}}}}}}}}}^{{\prime} }}{e}^{-{({{{{{{{\bf{r}}}}}}}}-{{{{{{{{\bf{r}}}}}}}}}^{{\prime} })}^{2}/2\pi {L}^{2}}{d}^{2}{{{{{{{{\bf{r}}}}}}}}}^{{\prime} }$$

Throughout the paper, we use *L* = 0.6*a*_0_ for all *R*-maps. The correlation lengths *ξ*_CDW_ and *ξ*_orient_ are then formed from the scalar fields ${A}_{x}({{{{{{{\bf{r}}}}}}}})=A({Q}_{x}^{*\ast },{{{{{{{\bf{r}}}}}}}})$ and ${A}_{y}({{{{{{{\bf{r}}}}}}}})=A({Q}_{y}^{*\ast },{{{{{{{\bf{r}}}}}}}})$ using two different methods as described in Refs. ^32,33^. Figure 1e summarizes the ratio of *ξ*_orient_/*ξ*_CDW_ obtained from each dataset. In every sample, both methods yield *ξ*_orient_/*ξ*_CDW_ > 0.5, revealing that Bi2201 tends more towards stripes than checkerboards, and that stripe order likely would also be present in the zero disorder limit. Regardless of whether the tendency to stripe modulations survives the hypothetical zero disorder limit, in the Bi2201 samples under consideration, our analyses show that there are local stripe domains present.

### Ising domains

**Fig. 2:**
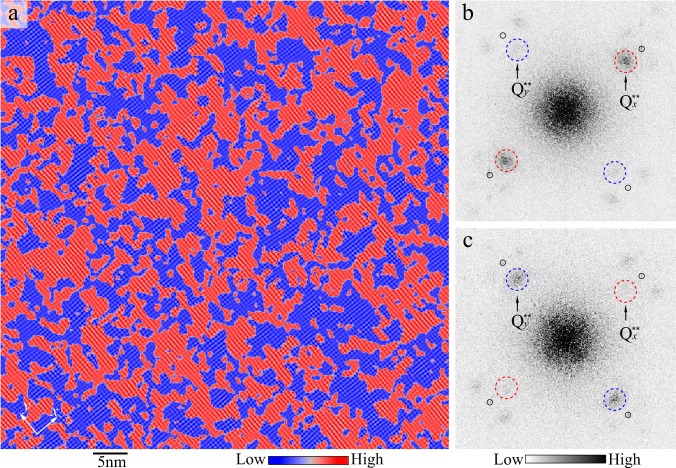
Mapping Ising domains. **a** A 65 nm × 65 nm Fourier-filtered *R*(**r**, 100 mV) map of UD32K, colored red ($\sigma=1,\, {Q}_{x}^{*\ast }$ dominates) and blue ($\sigma=-1,\, {Q}_{y}^{*\ast }$ dominates) to indicate the local Q^**^ unidirectional orientations. The unidirectional domains are derived from peaks around ${Q}_{x}^{*\ast }$ and ${Q}_{y}^{*\ast }$ with details in the text. The *R*-map has been Fourier-filtered to include only the power spectral density surrounding the four Q^**^ peaks (dashed circles in Fig. 1d). **b**, **c** Fourier transform of red- and blue-colored regions of non-filtered *R*-map in (**a**), respectively. ${Q}_{x}^{*\ast }$ dominates in the red regions (**b**), whereas ${Q}_{y}^{*\ast }$ in the blue regions (**c**).

Having identified the stripe nature of the local charge modulations in Bi2201, we map out where in the sample there are locally *x*-oriented domains, and where there are locally *y*-oriented domains. In Fig. 2, we show this mapping for the UD32K sample, constructed as follows: At each position **r**, the local FT *A*(**q**, **r**) is calculated according to Eqn. (1), which employs a Gaussian window of width *L*, with *L* optimized as described in the Supplementary information. We then integrate the FT intensity in a 2D gaussian window centered on ${{{{{{{\bf{q}}}}}}}}=\pm \!{Q}_{x}^{*\ast }$, and divide it by the integrated FT intensity around ${{{{{{{\bf{q}}}}}}}}=\pm \!{Q}_{y}^{*\ast }$. If this ratio is greater than a threshold *f* ~ 1 (i.e. ${Q}_{x}^{*\ast }$ is dominant), the region is colored red in Fig. 2a, otherwise the region is colored blue. The pattern thus derived in Fig. 2a is largely insensitive to changes in detail such as the exact center of the integration window, the size of the integration window, and the threshold *f* by which a cluster is colored. Similar results are also obtained in other samples with different chemical doping *p* by quantifying the FT intensity around Q^**^ and Q^*^. (See Supplementary Material.)

We analyze the pattern formation under the assumption that it is driven by a critical point under the superconducting dome. At the critical point of a second order phase transition, a system exhibits correlated fluctuations on all length scales, resulting in power law behavior for measurable quantities, with a different “critical exponent” controlling the power law of each quantity.

If the complex pattern formation shown in Fig. 2 is due to proximity to a critical point, then the critical exponents would be encoded in the geometric pattern, and the quantitative characteristics of the clusters would act like a fingerprint to identify the critical point controlling the pattern formation. This reveals information such as the relative importance of disorder and interactions. Because critical exponents are particularly sensitive to dimension, this analysis can also reveal whether the clusters form only on the surface of the material (like frost on a window), or whether they extend seamlessly from the surface into the bulk (like a tree whose roots reach deep underground). Unless the structures seen on the surface pervade the bulk of the material, they cannot be responsible for the bulk superconductivity.

### Critical exponents

**Fig. 3:**
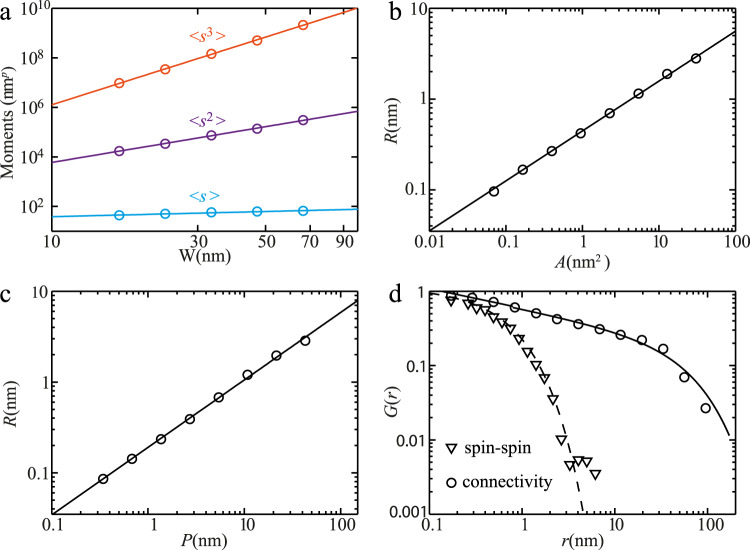
Cluster structure and power-law statistical analysis in UD32K sample. **a** Finite-size-scaling of moments for cluster size distribution, from which the Fisher exponent *τ* is calculated. Here p corresponds to the power indexes for the first (*p* = 1), second (*p* = 2) and third (*p* = 3) moments. **b** The radius of gyration *R* versus cluster area *A* showing a power law between them, from which the critical exponent ${d}_{v}^{*}$ is extracted. **c** The radius of gyration *R* versus effective cluster perimeter *P*. The perimeter *P* also shows a power law dependence on *R*, from which the critical exponent ${d}_{h}^{*}$ is extracted. **d** Spatial correlation functions *G*_conn_(**r**) (circles) and *G*_spin_(**r**) (triangles). The black line shows the best fit of the pair connectivity correlation function by ${G}_{{{{{{{{\rm{conn}}}}}}}}}\propto {r}^{-(d-2+{\eta }_{{{{{{{{\rm{pair}}}}}}}}})}\exp (-r/{\xi }_{{{{{{{{\rm{cluster}}}}}}}}})$, whereas the dashed line is only a guide to the eye. Logarithmic binning has been used in (**b**–**d**)^48^.

Near a critical point, the number of clusters *D* of a particular size *s* is power-law distributed, *D*(*s*) ∝ *s*^−*τ*^, where *s* is the number of sites in the cluster and *τ* is the Fisher critical exponent^34^. Figure 3a shows the first 〈*s*〉, second 〈*s*^2^〉, and third 〈*s*^3^〉 moments of the cluster size distribution as a function of the window size *W*, where *s* is the observed area of each cluster. Consistent with a system near criticality, the behavior of the moments vs. window size *W* displays robust power law behavior. The cluster moments are related to critical exponents by $\langle {s}^{n} \rangle \propto {W}^{(n+1 - \tau ){d}_{v}^{*}}$, where ${d}_{v}^{*}$ is the effective volume fractal dimension. Since the first moment $\langle {s} \rangle$ depends only weakly on *W* (leading to larger error in the estimate of the power law), we combine the information from 〈*s*^2^〉 and 〈*s*^3^〉 to derive *τ*. In the UD32K sample (Fig. 3a), we find *τ* = 1.97 ± 0.08.

The boundaries of clusters become fractal in the vicinity of a critical point, scaling as $H \propto {R}^{{d}_{h}}$ where *H* is the size of each cluster’s hull (outer perimeter), *R* is the radius of gyration of each cluster, and *d*_*h*_ is the fractal dimension of the hull. The interiors of the clusters also become fractal, scaling as $V \propto {R}^{{d}_{v}}$, where *d*_*v*_ is the volume fractal dimension of the clusters. Since STM probes only the sample surface, the observable quantities are the area $A \propto {R}^{{d}_{v}^{*}}$ and perimeter $P \propto {R}^{{d}_{h}^{*}}$ of each cluster, where ${d}_{v}^{*}$ and ${d}_{h}^{*}$ represent the effective volume and hull fractal dimension, respectively. In Fig. 3b and c, the cluster properties *A* and *P* are plotted vs. *R*, revealing a robust power law spanning 2.5 decades for both ${d}_{h}^{*}$ and ${d}_{v}^{*}$. Using a straightforward linear fit of the log-log plots (where the first point is omitted from the fit, since short-distance fluctuations are nonuniversal), we obtain the critical exponents ${d}_{v}^{*}=1.83\pm 0.08$ [Fig. 3b] and ${d}_{h}^{*}=1.36\pm 0.07$ [Fig. 3c].

We turn now to the orientation-orientation correlation function *G*_orient_(**r**), which is the analog of the spin-spin correlation function familiar from Ising models, *G*_orient_(**r**) = *G*_spin_(**r**) = 〈*S*(**r**)*S*(0)〉 − 〈*S*(0)〉^2^, where **r** = ∣**R**_*i*_ − **R**_*j*_∣ is the distance between (*x*, *y*) positions, here measured only on the surface. Near criticality, this function displays power law behavior as ${G}_{{{{{{{{\rm{orient}}}}}}}}}(r) \propto 1/{r}^{d-2+{\eta }_{||}}$, where *η*_∣∣_ is the anomalous dimension as measured at the surface, and *d* is the physical dimension of the phenomenon being studied, whether *d* = 2 for a surface phenomenon, or *d* = 3 for physics arising from the bulk interior of the material. Figure 3d shows *G*_orient_(**r**) for UD32K (triangles). For the UD32K sample as well as the other samples studied, *G*_orient_(**r**) does not have the standard power-law behavior expected near a critical point, but instead it decays more quickly with **r**.

Whereas the orientation-orientation correlation function is not power law in the data, the pair connectivity function, which is the probability that two aligned regions a distance *r* apart are in the same connected cluster^35^, does display robust power law behavior in the data, with ${G}_{{{{{{{{\rm{conn}}}}}}}}}(r) \propto {r}^{-(d-2+{\eta }_{{{{{{{{\rm{conn}}}}}}}}})}$, with *d* − 2 + *η*_conn_ = 0.29 +/− 0.036, as shown in Fig. 3d (circles).

While the pair connectivity function has been widely discussed for uncorrelated percolation fixed points^35^, where it is a power law, it has not previously been characterized at other fixed points. Our simulations of both the clean and random field Ising models show that the pair connectivity function is also a power law at the 2D clean Ising (C-2D) and the 2D random field Ising (RF-2D) fixed points (see Supplementary information). We find that it also displays power law behavior on interior 2D slices^36^ and at a free surface for the 3D clean Ising (C-3D) and 3D random field Ising (RF-3D) fixed points. In addition, our simulations of the clean and random field models close to but not at criticality show that there is a regime in which a short correlation length *ξ*_spin_ is evident in the spin-spin correlation function, in conjunction with robust power law behavior with a long correlation length *ξ*_cluster_ in the pair connectivity function, consistent with this dataset (see Supplementary information).

**Fig. 4:**
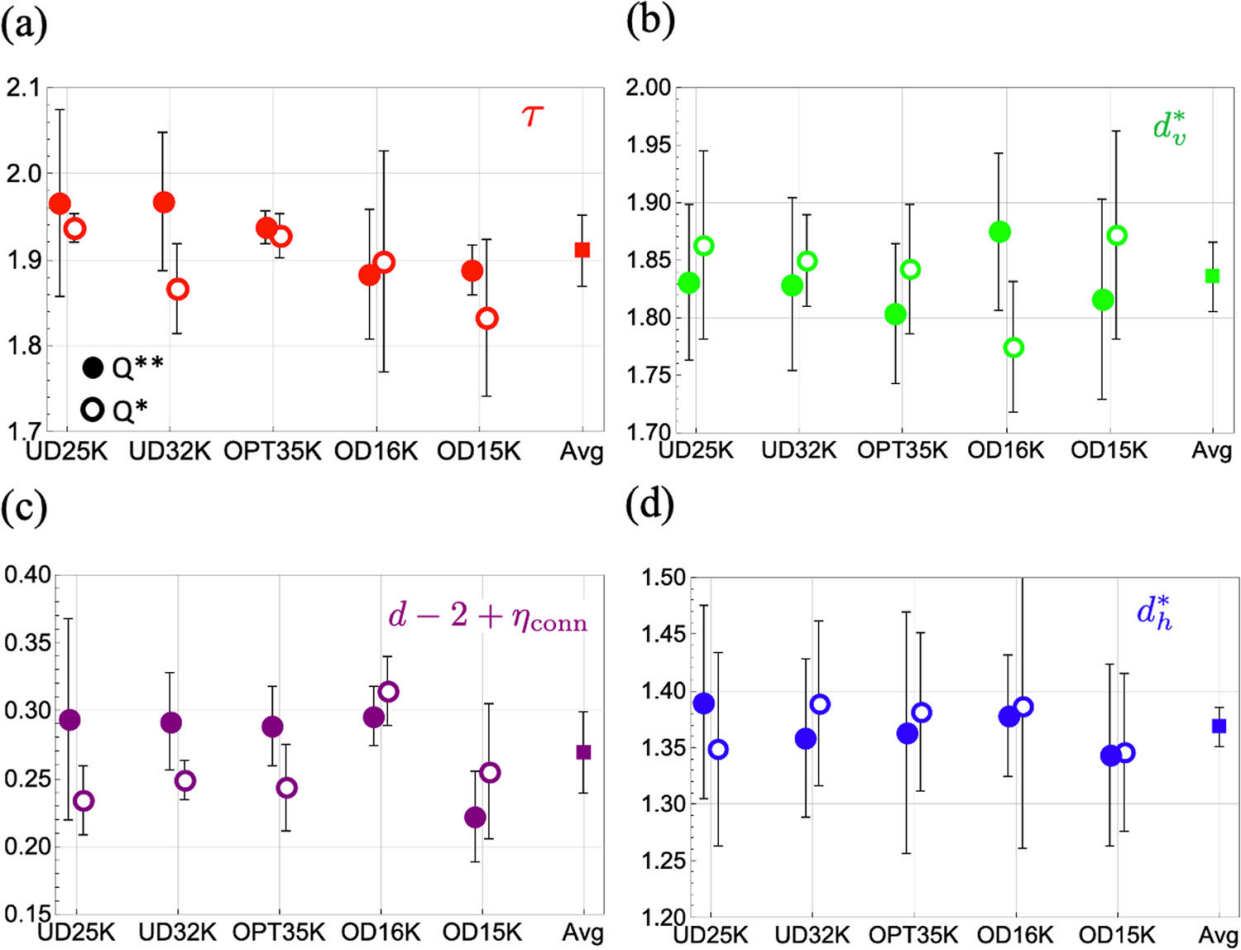
Critical exponents from experiment. Experimentally determined values of critical exponents derived from cluster maps based on Q^**^ (solid circles) and Q^*^ (open circles, see Supplementary information). **a** The Fisher exponent *τ*; **b** the volume fractal dimension ${d}_{v}^{*}$; **c** the combination *d* − 2 + *η*_conn_ where *η*_conn_ is the anomalous dimension of the pair connectivity function; and (**d**) the hull fractal dimension ${d}_{h}^{*}$. For the critical exponent *τ*, the error bars are estimated as the standard deviation of *τ* calculated from different gaussian width *L* and crop size W. For the critical exponents ${d}_{v}^{*}$, ${d}_{h}^{*}$, No obvious chemical doping dependence is observed, indicative of generic critical stripe correlations in Bi2201. Square symbols represent the average and standard deviation from cluster maps based on Q^**^ and on Q^*^ over all dopings.

**Fig. 5:**
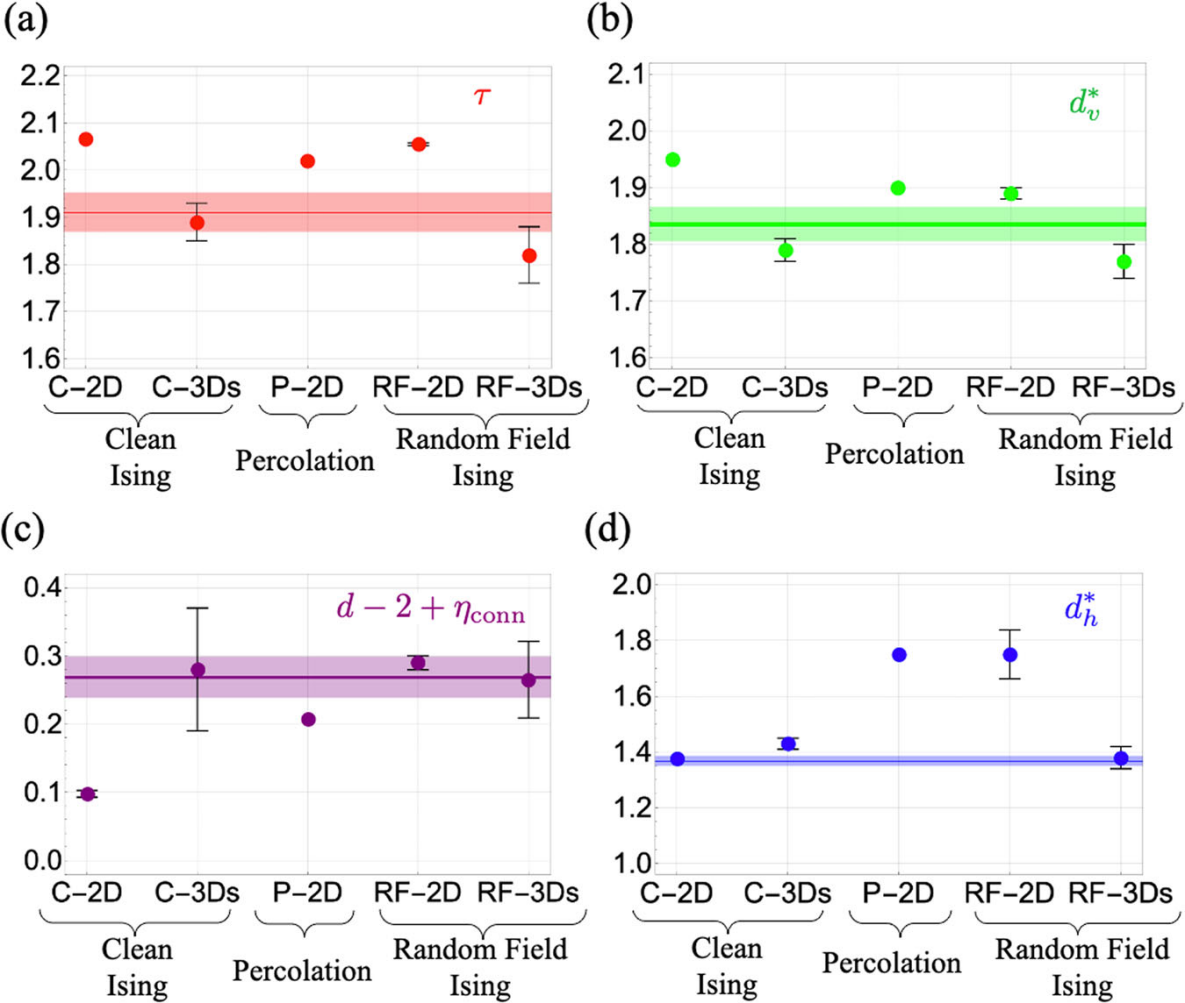
Critical exponent comparison. Comparison of experimentally determined exponents to theoretical values of exponents for candidate fixed points: **a** The Fisher exponent *τ*; **b** the volume fractal dimension ${d}_{v}^{*}$; **c** the combination *d* − 2 + *η*_conn_ where *η*_conn_ is the anomalous dimension of the pair connectivity function; and **d** the hull fractal dimension ${d}_{h}^{*}$. Circles represent theoretical values. Thin dark lines (thick transparent lines) are the average (standard deviation) of experimental values from cluster maps based on Q^**^ and on Q^*^ over all dopings.

Figure 4 shows the experimentally determined critical exponents for five dopings spanning from underdoped to overdoped, using cluster maps based on both Q^**^ and Q^*^. We find similar results at all dopings, which also show robust power laws, with the same exponents within error bars as the UD32K sample, and a cluster correlation length *ξ*_cluster_ which exceeds the FOV (see Supplementary information). Figure 5 shows a comparison between the theoretical critical exponents of Eqn. (2) and the experimentally determined values averaged over both Q** and Q* maps and over all dopings. The data-derived value of ${d}_{h}^{*}$ is inconsistent with the 2D percolation (P-2D) fixed point, indicating that interactions between stripe orientations must be present. In addition, the data-derived value for *d* − 2 + *η*_conn_ is inconsistent with that of the C-2D fixed point, and the data-derived value of ${d}_{h}^{*}$ is inconsistent with the RF-2D fixed point. The remaining candidate fixed points controlling the power law order of stripe orientations are C-3D and RF-3D (denoted C-3Ds and RF-3Ds, respectively, in the figure because we report theoretical values of the exponents at a free surface of the 3D models). Therefore, we find that the data-derived exponents are consistent with those of a layered clean or random field Ising model with *J*^⊥^ > 0 near criticality.

This shows that the fractal patterns observed here via STM are not confined to the surface, like frost growing on a window pane. Rather, these fractal stripe clusters fill the bulk of the material, more like a tree viewed through a 2D window. In the same way that transverse stripe fluctuations help electron pairs condense into a superconducting state rather than into the competing (insulating) pair crystal phase^37–39^, the stripe orientation fluctuations observed here could also have a profound effect on superconductivity, since orientation fluctuations of stripes also frustrate the pair crystal.

The doping independence evident in Fig. 4 is surprising, since one would expect there to be a phase transition from ordered to disordered stripe orientations as doping (a source of quenched disorder) is increased, with the critical, power law behavior observed here limited to the vicinity of the phase transition. While a broad region of critical behavior like that observed here is not natural near the C-3D fixed point, a broad region of critical behavior is characteristic of the RF-3D fixed point: for example, the cluster size distribution *D*(*S*) displays 2 decades of scaling, 50% away from the RF-3D critical point^40^. Therefore, the overall phenomenology, taking into account the cluster exponents, the static nature of the correlations, and the broad critical region, is consistent with the critical nematic correlations being controlled by the RF-3D fixed point.

## Discussion

While our findings suggest a prominent role for criticality in the phase diagram of cuprate superconductors, the spatial structures reported here are inconsistent with quantum criticality because these correlations are static on the timescales of several seconds, whereas quantum critical correlations fluctuate in time. In addition, for a quantum critical point tuned by doping, quantum critical scaling is confined to a narrow region close to the critical doping, in a “wedge” emanating from the critical doping and extending up in temperature. By contrast, we find critical, power law correlations at low temperature throughout the entire doping range measured. Whereas the lack of detectable doping dependence in finite FOV’s is inconsistent with quantum criticality, it is natural in the classical, three-dimensional random field Ising model. What the RF-3D fixed point shares in common with quantum criticality is that it is also a zero temperature critical point. However, it is tuned by disorder rather than by quantum fluctuations.

In addition, RF-3D is notoriously difficult to equilibrate in the vicinity of the critical point, since the relaxation time scales exponentially with the spin-spin correlation length: ${ \tau }_{{{{{{{{\rm{rel}}}}}}}}} \sim \exp [{\xi }_{{{{{{{{\rm{spin}}}}}}}}}^{\theta }]$, where the violation of hyperscaling exponent *θ* = 1.5. If the spin-spin correlation length reaches even 10 unit cells, the relaxation time will be $\exp [1{0}^{1.5}]\,\approx \, 5\times 1{0}^{13}$ times any bare microscopic timescale. Compare this with critical scaling near the C-3D fixed point, where for a correlation length of 10 unit cells, the relaxation time is on the order of ${ \tau }_{{{{{{{{\rm{rel}}}}}}}}} \propto {\xi }_{{{{{{{{\rm{spin}}}}}}}}}^{z}\,\approx \, 10$ times the bare timescale (where *z* is the dynamical critical exponent, which is of order 1).

As temperature is lowered on any given sample, it falls out of equilibrium if the relaxation time exceeds a timescale *t*_0_ which is set by the cooling protocol, ${ \tau }_{rel} \propto \exp [{\xi }_{{{{{{{{\rm{spin}}}}}}}}}^{\theta }]\,\gtrsim \, {t}_{0}$. Thus the orientational correlation length depends on the cooling protocol, rather than on doping, when approaching an ordered ground state (i.e. for doping *p* < *p*_*c*_), where the correlation length scales as *ξ*_spin_ ∝ 1/∣*T* − *T*_*c*_∣^*ν*^. Beyond that doping, the correlation length scales as *ξ*_spin_ ∝ 1/∣*p* − *p*_*c*_∣^*ν*^ at low temperature.

Our model unifies the observed power law scaling of stripe orientations in this material with the four decades of scaling seen in stripe orientations in NCCOC^19^. The fact that we see scaling all the way out to the FOV among four different critical exponents that are consistent with each other is strong evidence that the effect is due to criticality. If the scaling were to arise from some other mechanism, it is highly unlikely that all four exponents would line up with any theoretical model. Further tests of our model include the following: (1) At larger FOV, the cluster correlation length diverges as the critical doping is approached from large doping, as ${\xi }_{{{{{{{{\rm{cluster}}}}}}}}} \propto|p-{p}_{c}{|}^{-{\nu }_{{{{{{{{\rm{cluster}}}}}}}}}}$. Such a finding would also serve to identify the critical doping concentration for the vestigial nematic^41^. (2) The pair connectivity function will show a scaling collapse^40^ as a function of doping in the vicinity of the critical doping. (3) As a function of uniaxial in-plane strain, stripe orientations will experience switching events (avalanches) displaying Barkhausen noise^40^ in the vicinity of the critical point, typical of 3D RFIM criticality. (4) Finally, more fully aligned stripe orientations can be trained into the sample by cooling in the presence of in-plane strain^19^.

Our discovery that the charge modulations observed at the surface of Bi_2−*z*_Pb_*z*_Sr_2−*y*_La_*y*_CuO_6+*x*_ are locally one dimensional and also extend throughout the bulk of the material has important implications for the mechanism of superconductivity in cuprate superconductors. The fractal stripe clusters may have a profound effect on superconductivity, by frustrating competing orders like the pair crystal. The cluster analysis framework demonstrated here extends the capability of all surface probes used to study quantum materials to distinguish surface from bulk behavior. Furthermore, our finding that fractal stripe patterns both permeate the bulk of a cuprate superconductor and that they share universal features throughout the superconducting dome, raises important questions. Because doping naturally introduces disorder, a disorder-driven, zero temperature critical point for electronic nematicity is a very real possibility in other cuprates as well. Indeed, the fact that the CO in La_2−*x*_Sr_*x*_CuO_4_ is relatively unaffected by the onset of superconductivity^31^ may indicate that the superconducting dome in LSCO is far from a quantum critical point, as argued in Ref. ^42^, making it a good candidate to look for disorder-driven criticality as discussed here. More work is needed to further elucidate the connection between fractal electronic textures and superconductivity. For example, the connectivity correlation length of the stripes exceeds the field of view of our experiments throughout the doping range. An important open question for future studies is to establish the relationship between this correlation length and the optimal superconducting transition temperature.

## Methods

### STM measurements

Two different home-built STMs were used to acquire the data in this paper, both in cryogenic ultra-high vacuum. The samples were cleaved in situ at ~25 K and inserted immediately into the STM sample stage for imaging at 6 K. A mechanically cut polycrystalline PtIr tip was firstly calibrated in Au single crystals to eliminate large tip anisotropy. To obtain a tunneling current, we applied a bias to the sample while the tip was held at virtual ground. All tunneling spectra, which are proportional to the local density of states at given sample voltage, were measured using a standard lock-in technique.

### Theoretical models

Because there are only two orientations of the unidirectional domains, we can map the orientations to an Ising variable^19,43,44^, *σ* = ±1, where the + sign corresponds to red regions in Fig. 2, and the − sign corresponds to the blue regions. We model the tendency of neighboring unidirectional regions to align by a ferromagnetic interaction within each plane *J*^∣∣^ as well as an interlayer coupling *J*^⊥^

2 $$H=-\mathop{\sum}\limits_{ \langle ij{\rangle }_{||}}{J}^{||}{\sigma }_{i}{\sigma }_{j}-\mathop{\sum}\limits_{ \langle ij{\rangle }_{\perp }}{J}^{\perp }{\sigma }_{i}{\sigma }_{j}-\mathop{\sum}\limits_{i}({h}_{i}+h){\sigma }_{i}.$$

Any net orienting field, whether applied or intrinsic to the crystal, contributes to the bulk orienting field *h*^19^. In any given region, the local pattern of quenched disorder breaks the rotational symmetry of the host crystal, corresponding to random field disorder *h*_*i*_ in the Ising model. Quenched disorder can also introduce randomness in the couplings *J*, also known as random bond disorder. In the presence of both random bond and random field disorder, the critical behavior is controlled by the random field fixed point. In the model, *h*_*i*_ is chosen from a gaussian distribution of width Δ, which quantifies the disorder strength.

### Simulation methods

When comparing to a 2D model, the effective fractal dimensions observed at the surface can be compared directly with those of the model, ${d}_{v}^{*}={d}_{v},\, {d}_{h}^{*}={d}_{h}$. When comparing to a 3D model, we have calculated the cluster critical exponents of the model at a free surface, denoted C-3Ds and RF-3Ds in Fig. 5.

For the clean Ising model in three dimensions, because the fixed point (C-3D) controlling the continuous phase transition is at finite temperature, we use Monte Carlo simulations to generate stripe orientation configurations. To calculate the critical exponents of the 3D clean Ising model at a free surface, a 840 × 840 × 840 3D clean Ising model with periodic boundary conditions in the *x* and *y* direction and open boundary conditions in the *z* direction was simulated at *T*_*c*_ = 4.51*J* with 20,000 steps of the parallel Metropolis algorithm. To compare with the finite field of view of the experiments, we average over nine windows of size 256 × 256, taken from a free surface of the final spin configuration. The averages of these critical exponents are shown in Fig. 5. The standard deviations are smaller than the symbol size.

For the random field Ising model in three dimensions, the fixed point (RF-3D) controlling the continuous phase transition is at zero temperature, and we use a mapping to the max-flow min-cut algorithm^45–47^ to calculate exact ground state spin orientation configurations. The critical point of the 3D random field Ising model occurs at zero temperature. To calculate the 3D random field Ising model surface exponents, ground states were computed for 10 different disorder configurations of a 512 × 512 × 512 3D RFIM with open boundary conditions in the *z* direction and periodic boundary conditions in the *x* and *y* directions with *R* = 3, using a mapping between RFIM and the max-flow/min-cut problem^45–47^. To compare with the finite field of view of the experiments, the top surface of these ground states was windowed to system size 256 × 256 and critical exponents were extracted from the corresponding windows. The averages of these critical exponents are shown in Fig. 5. The standard deviations are smaller than the symbol size.

### Cluster methods

While the exponent *τ* derived from STM data is close to the narrow range allowed by the theoretical models, it is slightly below this range. Estimates of this exponent derived from a finite FOV are known to be skewed toward low values due to a bump in the scaling function, especially in the presence of random field effects^40^. To mitigate this effect, we perform finite-size scaling by analyzing the data as a function of window size *W*.

To mitigate possible window effects associated with a finite FOV in deriving the fractal dimensions *d*_*h*_ and *d*_*v*_, only the internal clusters that touch no edge of the Ising map have been included in the analyses of experimental data as well as simulation results. To extract the effective fractal dimensions, we adopt a standard logarithmic binning technique for analyzing power-law behavior^48^. (See also Supplementary Information and Refs. ^51–67^ therein.)

## Supplementary information

Supplementary Information

## Data Availability

All STM data presented here in the paper are available at 10.5281/zenodo.7682140.

## References

[1] Hoffman JE (2002). A four unit cell periodic pattern of quasi-particle states surrounding vortex cores in Bi_2_Sr_2_CaCu_2_O_8+*d*_. Science.

[2] Howald C, Eisaki H, Kaneko N, Kapitulnik A (2003). Coexistence of periodic modulation of quasiparticle states and superconductivity in Bi_2_Sr_2_CaCu_2_O_8+*d*_. Proc. Natl Acad. Sci. USA.

[3] Kohsaka Y (2007). An intrinsic bond-centered electronic glass with unidirectional domains in underdoped cuprates. Science.

[4] Wise WD (2008). Charge-density-wave origin of cuprate checkerboard visualized by scanning tunnelling microscopy. Nat. Phys..

[5] Hanaguri T (2004). A checkerboard electronic crystal state in lightly hole-doped Ca_2−*x*_Na_*x*_CuO_2_Cl_2_. Nature.

[6] Wu T (2011). Magnetic-field-induced charge-stripe order in the high-temperature superconductor YBa_2_Cu_3_O_*y*_. Nature.

[7] Chang J (2012). Direct observation of competition between superconductivity and charge density wave order in YBa_2_Cu_3_O_6.67_. Nat. Phys..

[8] Ghiringhelli G (2012). Long-range incommensurate charge fluctuations in (Y,Nd)Ba_2_Cu_3_O_6+*x*_. Science.

[9] Comin R (2014). Charge order driven by fermi-arc instability in Bi_2_Sr_2−*x*_La_*x*_CuO_6+*δ*_. Science.

[10] da Silva Neto EH (2015). Charge ordering in the electron-doped superconductor Nd_2−*x*_Ce_*x*_CuO_4_. Science.

[11] Croft TP, Lester C, Senn MS, Bombardi A, Hayden SM (2014). Charge density wave fluctuations in La_2−*x*_Sr_*x*_CuO_4_ and their competition with superconductivity. Phys. Rev. B.

[12] Tabis W (2014). Charge order and its connection with Fermi-liquid charge transport in a pristine high-*T*_*c*_ cuprate. Nat. Communi..

[13] Peng YY (2016). Direct observation of charge order in underdoped and optimally doped Bi_2_(Sr,La)_2_CuO_6+*δ*_ by resonant inelastic x-ray scattering. Phys. Rev. B.

[14] Kang M (2019). Evolution of charge order topology across a magnetic phase transition in cuprate superconductors. Nat. Phys..

[15] Li J (2020). Multiorbital charge-density wave excitations and concomitant phonon anomalies in Bi_2_Sr_2_LaCuO_6+*δ*_. Proc. Natl Acad. Sci. USA.

[16] Abbamonte P (2005). Spatially modulated ‘Mottness’ in La_2−*x*_Ba_*x*_CuO_4_. Nat. Phys..

[17] Comin R (2015). Symmetry of charge order in cuprates. Nat. Mater..

[18] da Silva Neto EH (2014). Ubiquitous interplay between charge ordering and high-temperature superconductivity in cuprates. Science.

[19] Phillabaum B, Carlson EW, Dahmen KA (2012). Spatial complexity due to bulk electronic nematicity in a superconducting underdoped cuprate. Nat. Commun..

[20] Lawler MJ (2010). Intra-unit-cell electronic nematicity of the high-*T*_*c*_ copper-oxide pseudogap states. Nature.

[21] Parker CV (2010). Fluctuating stripes at the onset of the pseudogap in the high-*T*_*c*_ superconductor Bi_2_Sr_2_CaCu_2_O_8+*d*_. Nature.

[22] Fujita K (2014). Direct phase-sensitive identification of a *d*-form factor density wave in underdoped cuprates. Proc. Natl Acad. Sci. USA.

[23] Tranquada JM, Sternlieb BJ, Axe JD, Nakamura Y, Uchida S (1995). Evidence for stripe correlations of spins and holes in copper oxide superconductors. Nature.

[24] Mook HA, Dai P, Dog F (1998). Spin fluctuations in YBa_2_Cu_3_O_6.6_. Nature.

[25] Comin R (2015). Broken translational and rotational symmetry via charge stripe order in underdoped YBa_2_Cu_3_O_6+*y*_. Science.

[26] Howald C, Eisaki H, Kaneko N, Greven M, Kapitulnik A (2003). Periodic density-of-states modulations in superconducting Bi_2_Sr_2_CaCu_2_O_8+*δ*_. Phys. Rev. B.

[27] Vershinin M (2004). Local ordering in the Pseudogap state of the high-*T*_*c*_ superconductor Bi_2_Sr_2_CaCu_2_O_8+*δ*_. Science.

[28] Arpaia R (2019). Dynamical charge density fluctuations pervading the phase diagram of a Cu-based high-*T*_*c*_ superconductor. Science.

[29] Gerber S (2015). Three-dimensional charge density wave order in YBa_2_Cu_3_O_6.67_ at high magnetic fields. Science.

[30] Jang H (2016). Ideal charge-density-wave order in the high-field state of superconducting YBCO. Proc. Natl Acad. Sci. USA.

[31] Wen J-J (2019). Observation of two types of charge-density-wave orders in superconducting La_2−*x*_Sr_*x*_CuO_4_. Nat. Commun..

[32] Robertson J, Kivelson S, Fradkin E, Fang A, Kapitulnik A (2006). Distinguishing patterns of charge order: Stripes or checkerboards. Phys. Rev. B.

[33] Del Maestro A, Rosenow B, Sachdev S (2006). From stripe to checkerboard ordering of charge-density waves on the square lattice in the presence of quenched disorder. Phys. Rev. B.

[34] Fisher ME (1967). The theory of condensation and the critical point. Phys. Phys. Fiz..

[35] Stauffer, D. & Aharony, A. *Introduction to Percolation Theory* (CRC Press, 1994).

[36] Liu S, Carlson EW, Dahmen KA (2021). Connecting complex electronic pattern formation to critical exponents. Condens. Matt..

[37] Carlson, E. W., Emery, V. J., Kivelson, S. A. & Orgad, D. *Concepts in High Temperature Superconductivity* (Springer-Verlag, 2004).

[38] Emery VJ, Kivelson SA, Zachar O (1997). Spin-gap proximity effect mechanism of high-temperature superconductivity. Phys. Rev. B.

[39] Kivelson SA, Fradkin E, Emery VJ (1998). Electronic liquid-crystal phases of a doped Mott insulator. Nature.

[40] Perković O, Dahmen K, Sethna J (1995). Avalanches, barkhausen noise, and plain old criticality. Phys. Rev. Lett..

[41] Nie L, Tarjus G, Kivelson SA (2014). Quenched disorder and vestigial nematicity in the pseudogap regime of the cuprates. Proc. Natl Acad. Sci. USA.

[42] Lee WS (2021). Spectroscopic fingerprint of charge order melting driven by quantum fluctuations in a cuprate. Nat. Phys..

[43] Carlson E, Dahmen KA, Fradkin E, Kivelson S (2006). Hysteresis and noise from electronic nematicity in high-temperature superconductors. Phys. Rev. Lett..

[44] Carlson EW, Liu S, Phillabaum B, Dahmen KA (2015). Decoding spatial complexity in strongly correlated electronic systems. J. Supercond. Nov. Magn..

[45] Elias P, Feinstein A, Shannon C (1956). A note on the maximum flow through a network. IRE Trans. Inf. Theory.

[46] Goldberg, A. V. In *Algorithmic Aspects in Information and Management, Lecture Notes in Computer Science*, 212–225 (Springer-Verlag, Berlin, Heidelberg, 2009).

[47] Picard JC, Ratliff HD (1975). Minimum cuts and related problems. Networks.

[48] Newman MEJ (2005). Power laws, Pareto distributions and Zipf’s law. Contemp. Phys..

[49] Hacker, T., Yang, B. & McCartney, G. *Empowering Faculty: A Campus Cyberinfrastructure Strategy for Research Communities* (Educause, 2014).

[50] Presland M, Tallon J, Buckley R, Liu R, Flower N (1991). General trends in oxygen stoichiometry effects on *T*_*c*_ in Bi and Tl superconductors. Phys. C: Supercond..

[51] Kohsaka Y (2008). How Cooper pairs vanish approaching the Mott insulator in Bi_2_Sr_2_CaCu_2_O_8+d_. Nature.

[52] He Y (2014). Fermi surface and pseudogap evolution in a cuprate superconductor. Science.

[53] Hanaguri T (2007). Quasiparticle interference and superconducting gap in Ca_2−x_Na_x_CuO_2_Cl_2_.. Nature Physics.

[54] Cardy, J. *Scaling and Renormalization in Statistical Physics*. (Cambridge University Press, Cambridge 1996).

[55] Janke W, Schakel AMJ (2005). Fractal structure of spin clusters and domain walls in the two-dimensional Ising model. Phys. Rev. E.

[56] Stauffer, D. & Aharony, A. *Introduction To Percolation Theory*. (Taylor & Francis, 2018).

[57] Stauffer D (1979). Scaling theory of percolation clusters. Physics Reports.

[58] Grossman T, Aharony A (1999). Accessible external perimeters of percolation clusters. J. Phys. A Math. Gen..

[59] Környei L, Iglói F (2007). Geometrical clusters in two-dimensional random-field Ising models. Phys. Rev. E.

[60] Ji H, Robbins MO (1991). Transition from compact to self-similar growth in disordered systems: Fluid invasion and magnetic-domain growth. Phys. Rev. A.

[61] Seppälä ET, Alava MJ (2001). Susceptibility and percolation in two-dimensional random field Ising magnets. Phys. Rev. E.

[62] Drossel B, Dahmen K (1998). Depinning of a domain wall in the 2d random-field Ising model. The European Physical Journal B - Condensed Matter and Complex Systems.

[63] Stauffer D (1979). Scaling theory of percolation clusters. Physics Reports.

[64] Janke W, Schakel AMJ (2005). Fractal structure of spin clusters and domain walls in the two-dimensional Ising model. Physical Review E.

[65] Saberi AA, Dashti-Naserabadi H (2010). Three Dimensional Ising Model, Percolation Theory and Conformal Invariance. Europhys Lett..

[66] Livet F (1991). The Cluster Updating Monte Carlo Algorithm Applied to the 3d Ising Problem. Europhys Lett..

[67] Talapov AL, Bl¨ote HWJ (1996). The magnetization of the 3D Ising model. J. Phys. A Math. Gen..

