# Supplementary material for: Critical nematic correlations throughout the superconducting doping range in Bi2−zPbzSr2−yLayCuO6+x
Source: Nat Commun. 2023 May 5;14:2622. doi: 10.1038/s41467-023-38249-3 (PMC10162959; doi:10.1038/s41467-023-38249-3)
Supplement: Supplementary file 1 — Supplementary Information [file 41467_2023_38249_MOESM1_ESM.pdf]

Supplementary Material for

Critical nematic correlations throughout the superconducting doping range in $Bi_{2-z}Pb_zSr_{2-y}La_yCuO_{6+x}$

A. Relationship between Q^* and Q^{**}

While $\sim 4a_0$ charge order (wavevector $Q^* \sim 2\pi/4a_0$) has long been observed in a number of cuprates [1, 2, 4, 5], and the finer structure of charge localization on oxygen p orbitals within each $\sim 4a_0$ plaquette (longer wavevector $Q^{**} \sim 2\pi(3/4a_0)$) has also been noted [3, 5, 21], the exact relationship between Q^* and Q^{**} was clarified more recently as a d-form-factor density wave [22]. Thus $Q^* + Q^{**} = 1$, as we confirm in Fig. S1. However, the longer-wavelength structure (corresponding to Q^*) manifests primarily at low energies within the pseudogap, while the finer structure (corresponding to Q^{**}) manifests primarily at high energies above the pseudogap scale (e.g. see Supplementary Figs. S5 in Ref. [51] and S5 in Ref. [21]).

Following convention, we quantify Q^{**} using the R-map, where $R(\mathbf{r}, V) = I(\mathbf{r}, V)/I(\mathbf{r}, -V)$, and $I(\mathbf{r}, \pm V)$ represents the STM tunneling current at $\pm V$ as a function of position $\mathbf{r}$ on the surface of the sample [3].

$$R(\mathbf{r}) \equiv \frac{\int_0^{\Omega_c} N(\mathbf{r}, E)dE}{\int_{-\infty}^{0} N(\mathbf{r}, E)dE} \approx \frac{\int_0^{V} dI/dV'(\mathbf{r}, +V')dV'}{\int_{-V}^{0} dI/dV'(\mathbf{r}, -V')dV'} = \frac{I(\mathbf{r}, +V)}{I(\mathbf{r}, -V)}, \tag{S1}$$

where Ω_c satisfies "all low-energy scales" $< \Omega_c <$ Hubbard U. Thus the integration limit V must be larger than the pseudogap energy scale, which varies from ~ 60 mV for the UD25K sample down to ~ 20 mV for the OD15K sample [52]. For experimental efficiency, we chose $V = 100mV$ for UD25K and UD32K, $V = 80$ mV for OPT35K, and at least $V = 45$ mV for OD16K and OD15K samples.

Becuase Q^* is most pronounced at subgap energies, we quantify Q^* by summing dI/dV from -20 mV to 20 mV, hereafter denoted as an S-map,

$$S(\mathbf{r}) \equiv \int_{-20\text{mV}}^{+20\text{mV}} dI/dV'(\mathbf{r}, V')dV'. \tag{S2}$$

Our S-map is similar to the definition in Ref. [21], albeit slightly different normalization. We note that within the superconducting gap, dispersing Bogoliubov quasiparticle interference (QPI) is also present, but the QPI signal is anti-symmetric about E_F [53], so our choice to integrate over both negative and positive energies will cancel the QPI and enhance the Q^* signal in the S-map.

B. Setting the gaussian window of the FT

In applying Eqn. 1 to the data, the width of the gaussian window of the FT is set by the parameter L. In order to find the optimal L, we define a quality factor $H = \mathrm{F}_x + \mathrm{F}_y^{'} - \mathrm{F}_y - \mathrm{F}_x^{'}$. Here F_x and F_y represent the integrated FT intensity around $\pm Q_x^{**}$ and $\pm Q_y^{**}$ of the red-colored regions of raw R-map ($\sigma = +1$, Fig. 2(b)), while $\mathrm{F}_x^{'}$ and $\mathrm{F}_y^{'}$ the integrated FT intensity around $\pm Q_x^{**}$ and $\pm Q_y^{**}$ of the blue-colored regions of raw R-map ($\sigma = -1$, Fig. 2(c)). A larger H value means a higher quality of the Ising map. We therefore choose the optimal L and f by maximizing H. In our study, we find that L is typically in the range of (0.4-0.9)a_0. For consistency, we have chosen $L = 0.6a_0$ for all R-maps in Fig. 2(a) and Supplementary Fig. S2(a-e). We choose $L = 0.9a_0$ for all S-maps in Fig. S2(f-j).

C. Critical Points of the Models

At a critical point, a system displays critical, power law behavior at all length scales. Near but not at a critical point, a system displays critical behavior below a finite correlation length ξ, which approaches infinity at the critical point. The critical fixed points which control the continuous phase transitions contained in the models of Eqn. 2 are as follows: With no quenched disorder ($\Delta = 0$), the universality class is that of the clean Ising model. The critical exponents are different in two dimensions (at the C-2D fixed point), *vs.* three dimensions (at the C-3D fixed point). In the noninteracting limit $J_{||} = J_\perp = 0$, Eqn. 2 describes the percolation model, in which the stripe orientation at each site $\sigma_i = \pm 1$ takes a value independently of its neighbor. The only time this model can display criticality when viewed on a two-dimensional FOV is when the probability of having, say, $\sigma_i = +1$ on each site is near $p = 0.59$,

(or its complement $p = 1 - 0.59$) *i.e.* at P-2D. When both interactions and random field disorder are present, the universality class is that of the three-dimensional random field Ising model (RF-3D) when $J_\perp > 0$, or that of RF-2D if $J_\perp = 0$. Any finite coupling $J^\perp > 0$ in the third dimension is relevant in the renormalization group sense, even in a strongly layered model with $J^\perp \ll J^{||}$, so that ultimately the critical behavior is that of the 3D model at long enough length scales, whether clean (C-3D) or random field (RF-3D).

Table SI. Theoretical Critical Cluster Exponents of Two Dimensional Ising Models

Model	Clean Ising Model	Percolation	Random Field Disorder
Critical Exponent	C-2D	P-2D	RF-2D
τ	2.067[54, 55]	2.02 [56–58]	$2.055 \pm .003$ [59]
d_v	187/96=1.95[54, 55]	91/48=1.9 [56–58]	$1.89 \pm .01$ [60]
d_h	11/8=1.375[54, 55]	7/4=1.75[56–58]	$1.75 \pm .009$ [60–62]
η_{conn}	$0.098 \pm .005$ (this work)	0.207 [56–58]	$0.29 \pm .01$ (this work)

Table SII. Theoretical Critical Cluster Exponents of Free Surfaces of Three Dimensional Ising Models

Model	Clean Ising Model	Random Field Disorder
Critical Exponent	C-3Ds	RF-3Ds
τ	$1.89 \pm .04$ (this work)	$1.82 \pm .06$ (this work)
d_v	$1.79 \pm .02$ (this work)	$1.77 \pm .03$ (this work)
d_h	$1.43 \pm .02$ (this work)	$1.38 \pm .04$ (this work)
η_{conn}	$0.28 \pm .09$ (this work)	$0.265 \pm .06$ (this work)

D. Comparing data-derived critical exponents to theoretical models

As evident in the data, the nematic orientation-orientation correlation function (spin-spin correlation function in the Ising language) does not display robust power law behavior. On the other hand, the connectivity function (defined as the probability that two aligned sites are connected by the same cluster) does show robust power law behavior. Because this behavior is known to be qualitatively consistent with uncorrelated percolation fixed points [63], one might surmise that the intricate pattern formation observed on the surface of this set of materials is simply due to uncorrelated percolation, which would lead to the dubious conclusion that the role of *both* interactions and disorder is *irrelevant* in the renormalization group sense for the multiscale pattern formation in this material. While the extracted exponent for the anomalous dimension of the connectivity function $d - 2 + \eta_{\text{connect}}$ is somewhat close to that of uncorrelated 2D percolation, the hull fractal dimension in that model is $d_h = 7/4 = 1.75$ [64], which is contradicted by the experimental data shown here. Note that 3D percolation at criticality is also ruled out, not by the large discrepancy between the theoretical and data-derived values of the exponent $d - 2 + \eta_{\text{connect}}$, but rather by the fact that the clusters do not display fractal properties at a free surface at the 3D percolation fixed point. This is underscored by the fact that the connectivity function in that model is power law in the bulk, but is not power law on a 2D slice. Therefore, the pattern formation in these Bi2201 samples is *not* controlled by uncorrelated percolation, but there must exist some other fixed point(s) at which the connectivity function is power law.

Our Monte Carlo simulations of the clean 2D Ising model at the critical temperature reveal that in fact, the connectivity function is also power law at the clean 2D fixed point, with an exponent $d - 2 + \eta_{\text{connect}} = 0.098 \pm .005$, which also serves to rule out the clean 2D fixed point as the origin of this behavior in the data [36]. Note that the exponents associated with the geometric clusters at the C-2D fixed point are $\beta_p/\nu_p = 5/96$ and $d_{v,p} = 187/96$ [64], where the volume fractal dimension $d_{v,p}$ is derived from the fractal structure of the geometric clusters as they percolate at $T_p = T_c$. Inserting these values into the exponent relation $2\beta_p/\nu_p = 2(d - d_{v,p})$ yields $2(5/96) = 2(2 - 187/96)$, indicating that the geometric clusters also satisfy scaling relations at C-2D.

The behavior in question concerns the observed correlation functions on a free surface of the material, rather than the bulk correlation functions. Our simulations of both the clean Ising model and the random field Ising model in three dimensions reveal that in fact, the connectivity function *at a free surface* displays power law behavior at the thermodynamic critical point. (See Fig. S10 and Ref. S11.) For the clean model, this behavior is directly related to the

fact that while 3D geometric clusters do not undergo a (correlated) percolation point until $T_p < T_c$, geometric clusters on a 2D slice undergo a (correlated) percolation point right at $T_p^{\text{2D slice}} = T_c$ [36, 65], indicating that the geometric clusters on the surface also undergo a (correlated) percolation point at the same temperature. Our results indicate that in the same way, geometric clusters on the free surface of a 3D random field model also undergo a (correlated) percolation point at $T_p^{\text{2D slice}}$, leading to the robust power law connectivity function revealed in our simulations of the 3D random field Ising model. Our results for the critical exponents at a free surface of the clean and random field models in three dimensions are shown in Fig. 5(c), demonstrating a good match between the data and either a clean or random field 3D Ising model for this measure.

We have also calculated the connectivity function of the 2D random field Ising model. For weak random field strength, we find that the connectivity function is also a power law in this model, with exponent $d - 2 + \eta_{\text{connect}} \approx 0.29 \pm 0.01$.

The pair connectivity function and the spin-spin correlation function are plotted in Fig. S10 for one disorder configuration, on a 256×256 window on the open surface of the three-dimensional random field Ising model of system size $512 \times 512 \times 512$ with open boundary conditions in the z direction and periodic boundary conditions in the x and y directions. Both plots are logarithmically binned and fit to a function of the form $G(r) = Ce^{-r/\xi} r^{-(d-2+\eta)}$. In the spin-spin correlation plot, for several of the computed points starting with $r \approx 11.4$, the measured value of $G(r)$ is negative. These negative points cannot be displayed on a log scale and cannot be included in the exponentially decaying power law fit. The downward trend in $G(r)$ followed by values fluctuating around 0 indicate that the error in the measured values due to finite size effects is of similar size to the values themselves. Thus, all values after $r = 11$ are excluded from the fit. Note that while the spin-spin correlation function is not robustly power law, the pair connectivity function is. The same behavior appears in the experimental data as shown Fig. 3(d). While it is well-known that the pair connectivity function is power law near criticality in uncorrelated percolation, it was not previously known that the pair connectivity function (defined on a surface) is power law near criticality of the 3D RFIM.

The pair connectivity and spin-spin correlation function are plotted in Fig. S11 for a 256×256 window on a free surface of a clean 3D Ising model of system size $840 \times 840 \times 840$ with open boundary conditions in the z direction and periodic boundary conditions in the x and y directions simulated at $T = T_c$. Both plots are logarithmically binned and fit to a function of the form $G(r) = Ce^{-r/\xi} r^{-(d-2+\eta)}$. In the spin-spin correlation plot, for several of the computed points starting with $r \approx 38.4$, the measured value of $G(r)$ is negative. Because these points cannot be included in te fit, all subsequent points have also been omitted. While the spin-spin correlation function is not robustly power law, the pair connectivity is.

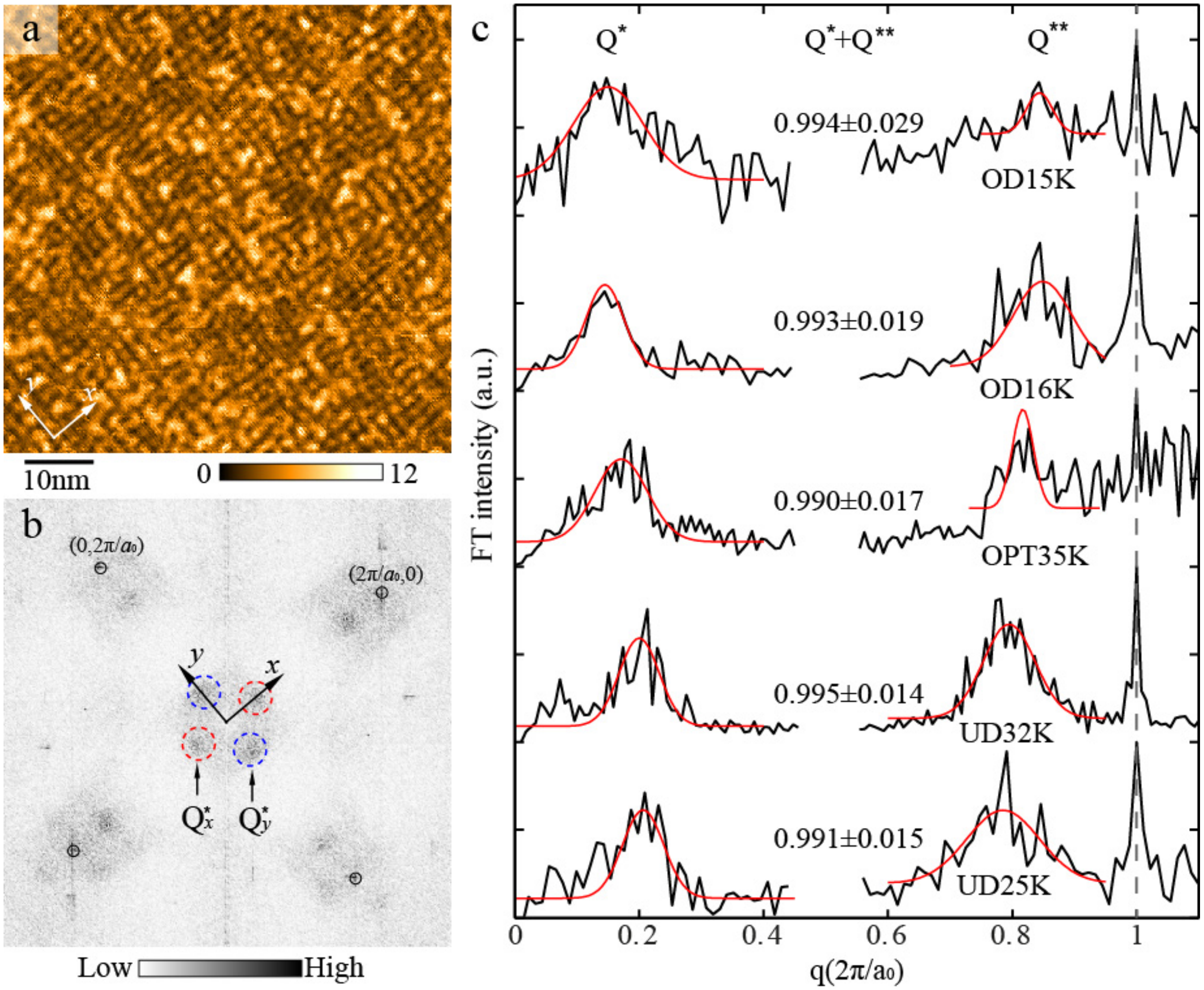

Figure S1. **The sum of wavevector $Q^* + Q^{**} \simeq 1$.** (**a**) Map of dI/dV integrated over the energy range of -20 mV and 20 mV (S-map, as defined in Eq. S2), acquired in the same field of view shown in Fig. 2a. The $\sim 4a_0$ charge modulation is quite prominent in real space. The conductance dI/dV maps are acquired at $I = 400$ pA and $V_s = -200$ mV. (**b**) Fourier transform of S map in (**a**), with Bragg vectors $(\pm1,\, 0)2\pi/a_0$ and $(0,\, \pm1)2\pi/a_0$ marked by black circles. The wavevectors $Q_x^* \sim (1/4,\, 0)2\pi/a_0$ and $Q_y^* \sim (0,\, 1/4)2\pi/a_0$ from the charge modulation are pronounced and marked by dashed circles and arrows, from which Q* wavevectors are extracted. The central broad FT intensities have been eliminated by subtracting the radial background, obtained by a power-law fit of the FT intensities near the diagonal $x \pm y$ directions. (**c**) Gaussian fits (red curves) of FT intensities in various samples. The left and right black curves are extracted from the fourier-transformed images of S and R-maps, respectively. Note that $Q^* + Q^{**} \simeq 1$ holds for all samples. The errors indicate the standard derivation of $Q^* + Q^{**}$ values obtained by choosing different wavevector windows for the Gaussian fits.

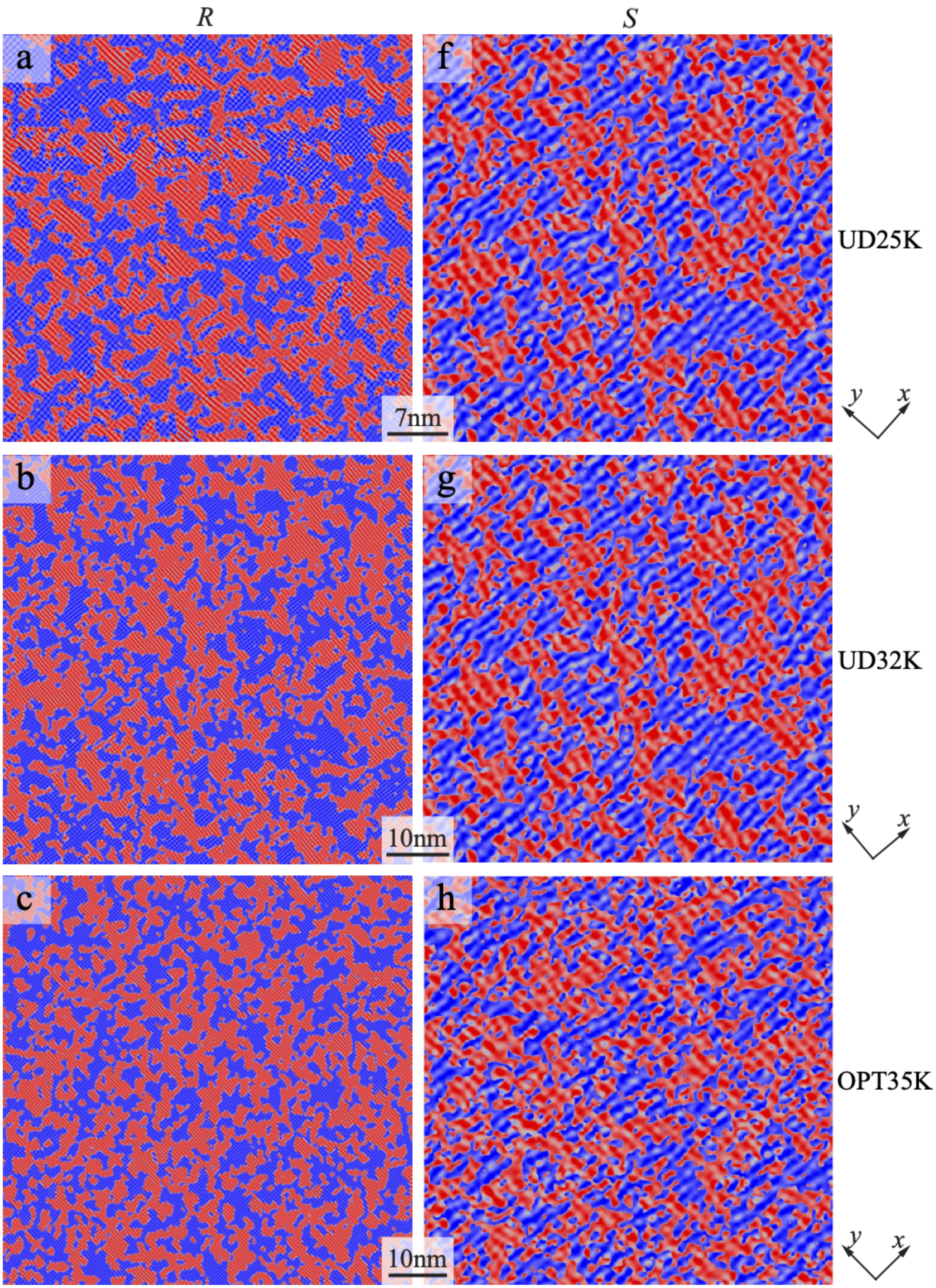

Figure S2. **Ising domains.** See caption on next page.

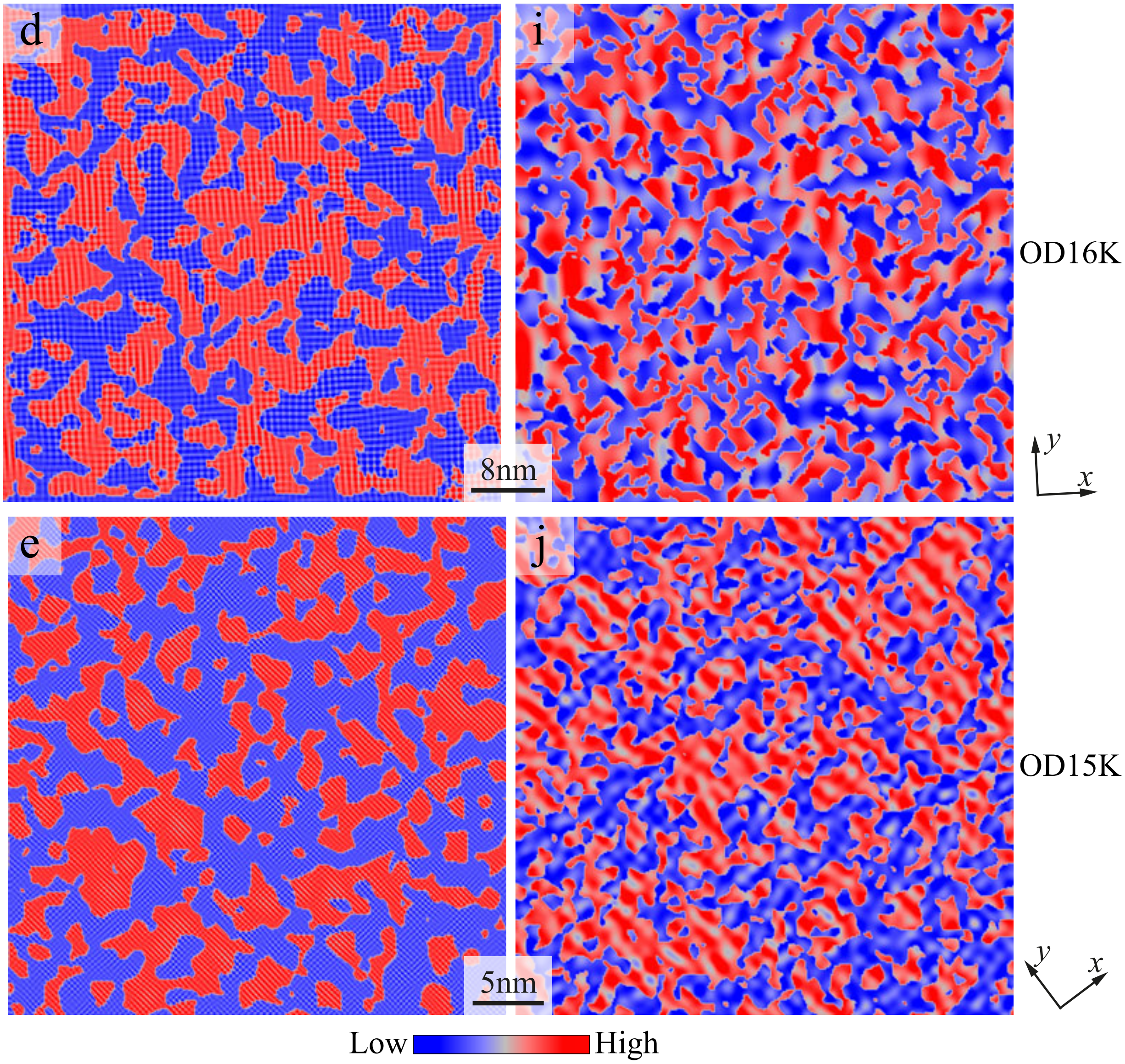

Figure S2. **Ising domains.** (**a-j**) Mapping Ising domains from R-maps (**a-e**) and S-maps (**f-j**) in various samples, colored red ($\sigma = 1$, $\mathrm{Q}_x^{**}/\mathrm{Q}_x^*$ dominates) and blue ($\sigma = -1$, $\mathrm{Q}_y^{**}/\mathrm{Q}_y^*$ dominates) to indicate the local unidirectional orientations. We map the Ising domains in (**f-j**) by comparing the integrated FT intensity between around $\mathbf{q} = \pm Q_x^*$ and $\mathbf{q} = \pm Q_y^*$ using a similar round integration window centered at $\pm Q_x^*$ and $\pm Q_y^*$ (dashed circles in Supplementary Fig. S1b). The Gaussian width $L = 0.9a_0$ has been chosen to calculate the Ising maps from S-map datasets (**f-j**). For clarity, all maps have been fourier-filtered to include only the FT power spectral density surrounding Q^{**} (**a-e**) or Q^* (**f-j**) peaks.

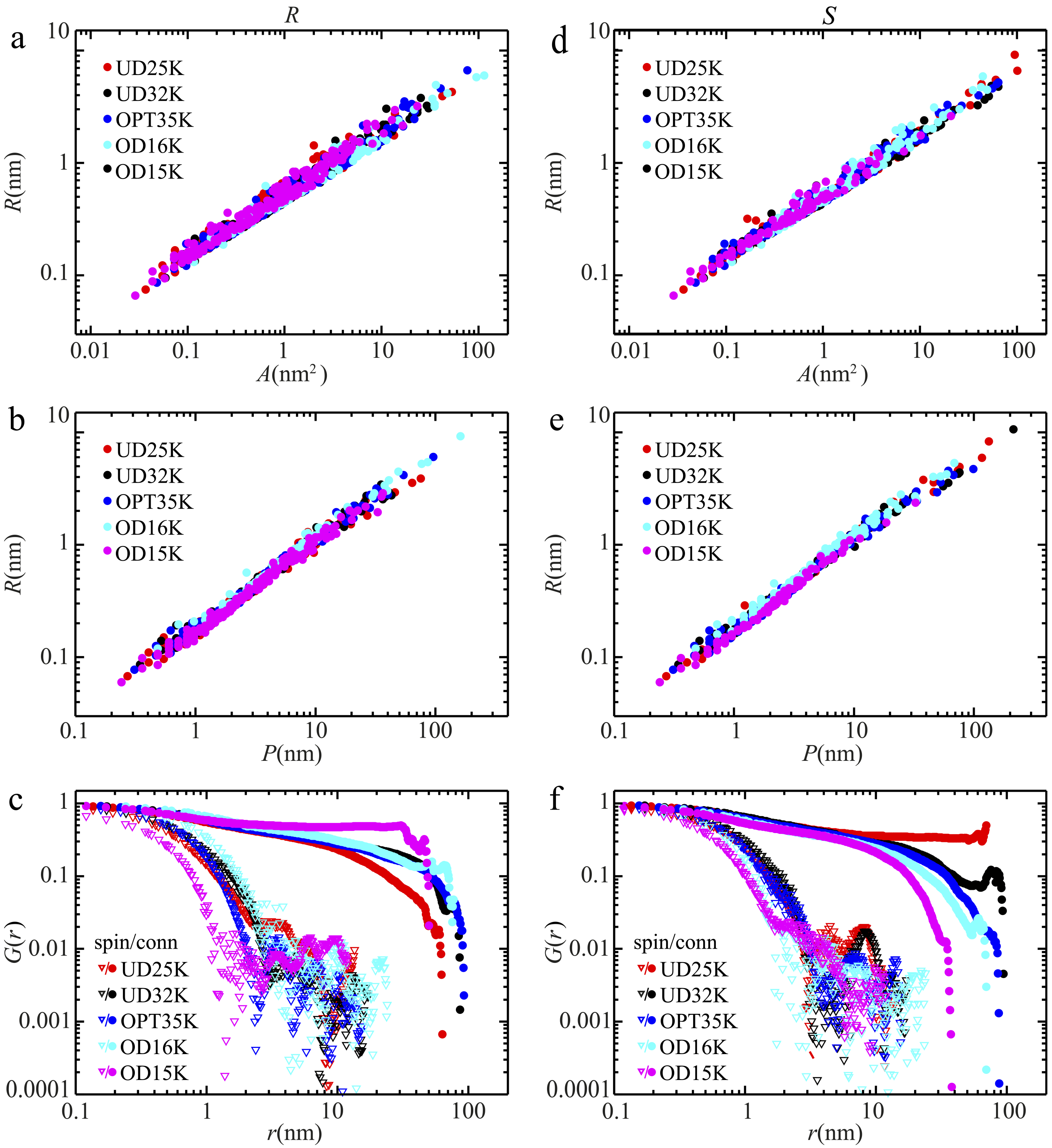

Figure S3. **Cluster structure and correlation functions.** Statistics and correlation functions of Ising cluster derived from R-maps (**a-c**) and S maps (**d-f**) in various samples. The empty triangles and solid circles in (**c**) and (**f**) indicate the spatial spin-spin and connectivity correlation functions, respectively.

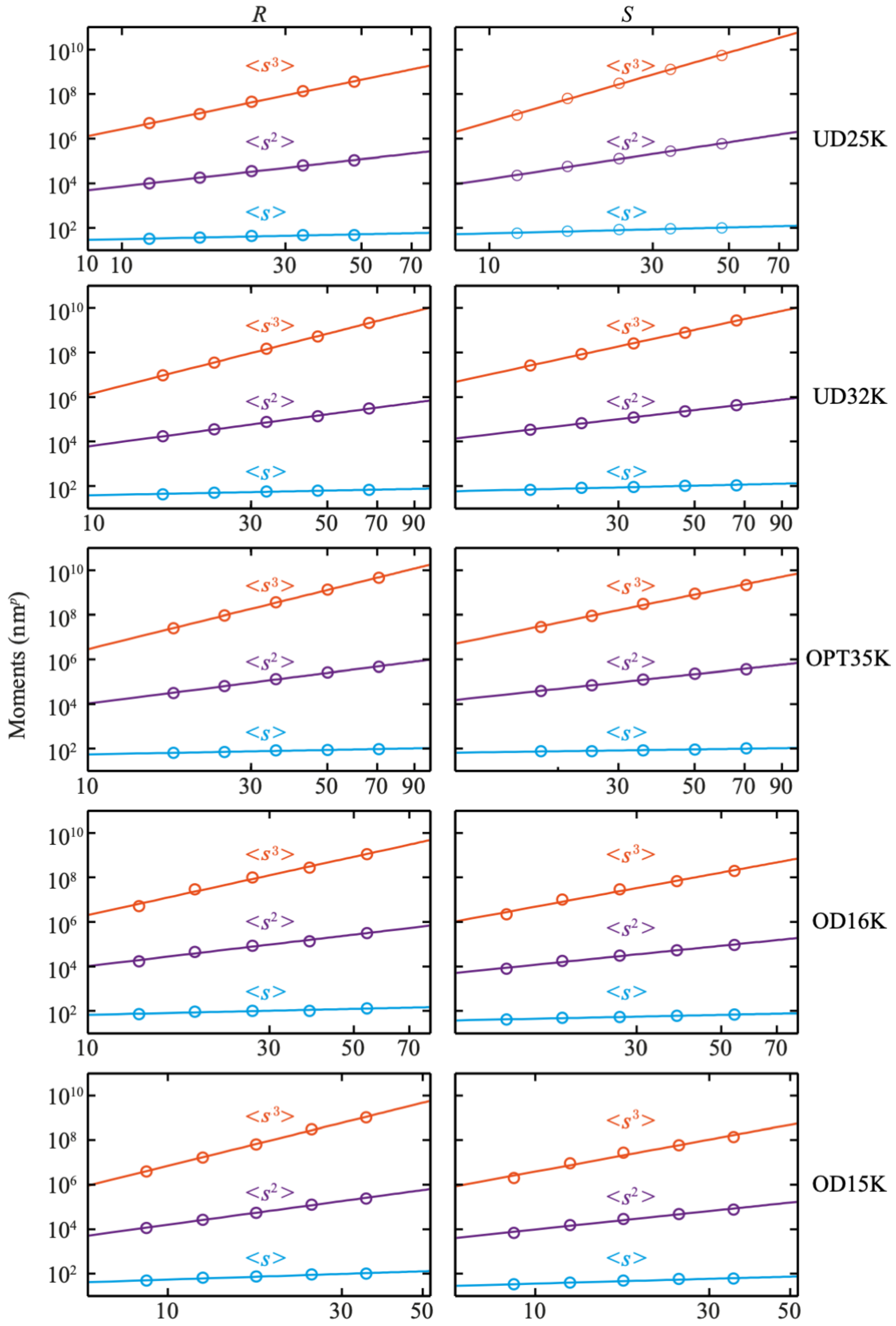

Figure S4. **Finite-size-scaling of moments for cluster size distribution.** A power law is generally found between each moment and crop size W for all Ising maps in Supplementary Fig. S2, either from R (**a-e**) and S-maps (**f-j**).

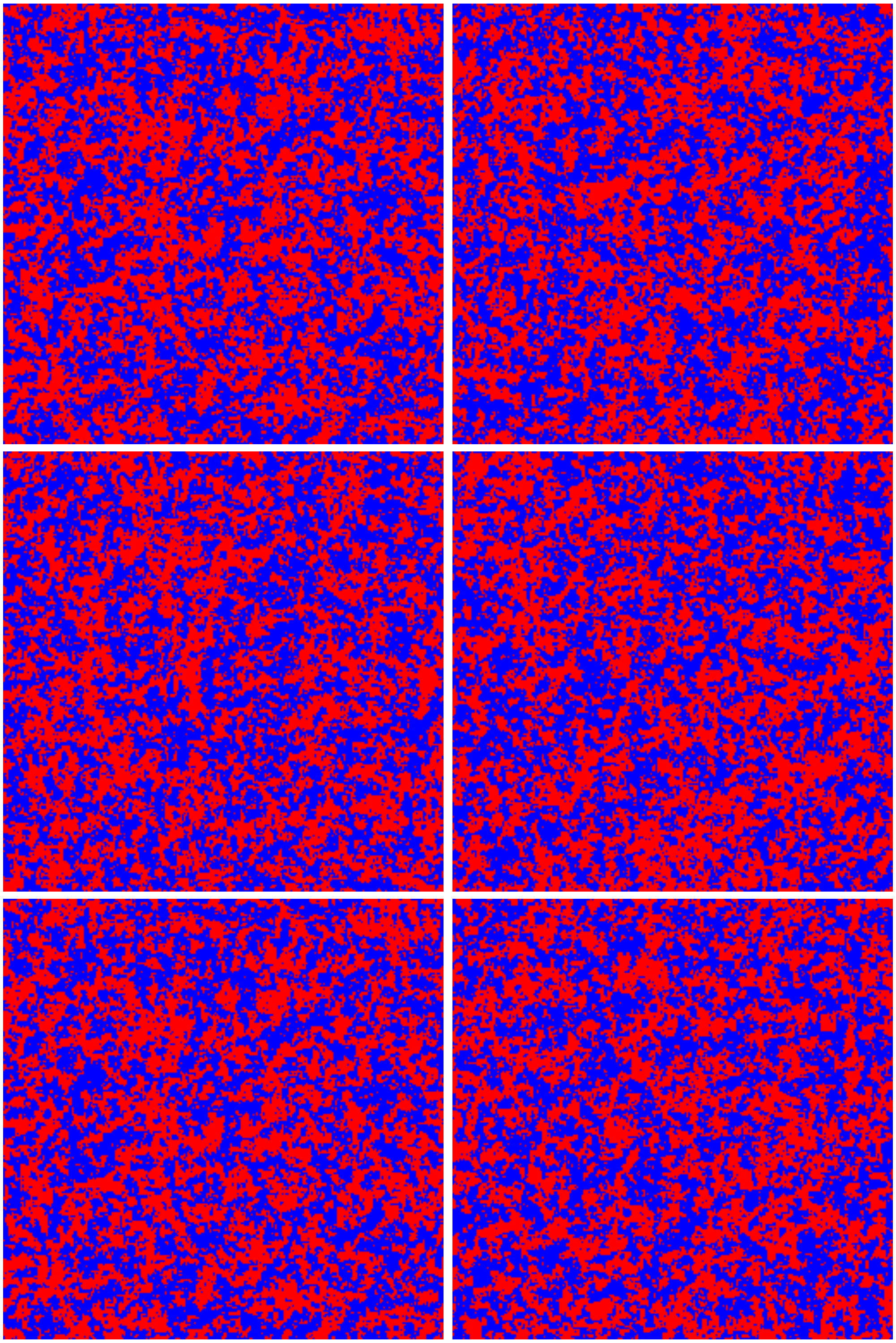

Figure S5. Representative ground state configurations of stripe orientations on a free surface of the near-critical 3D RFIM at disorder strength $R = 3J$. The images show windows of size 256×256 on the free surface taken from exact calculations of the ground state of systems of size $512 \times 512 \times 512$.

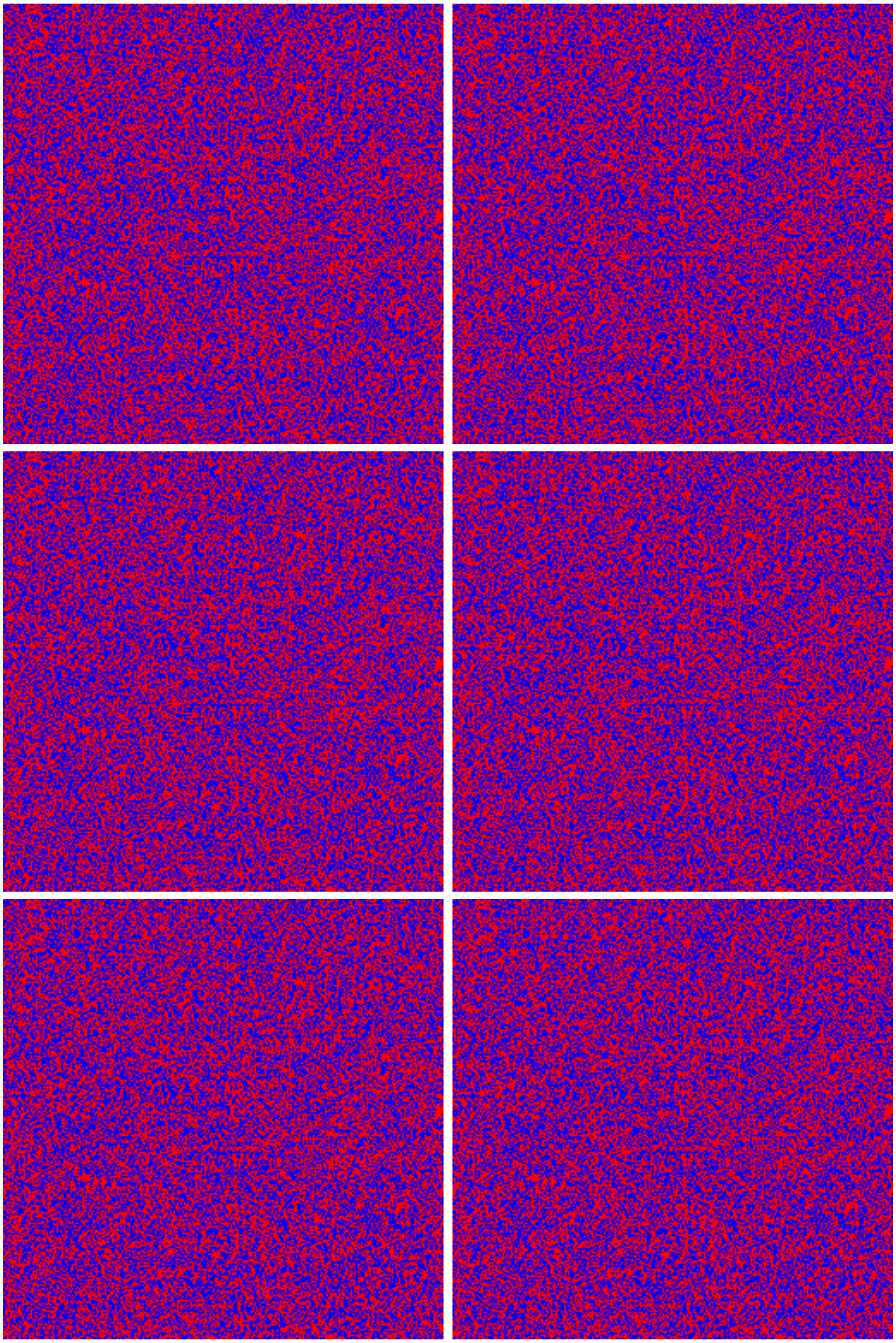

Figure S6. 256×256 percolation images at $p = p_c$

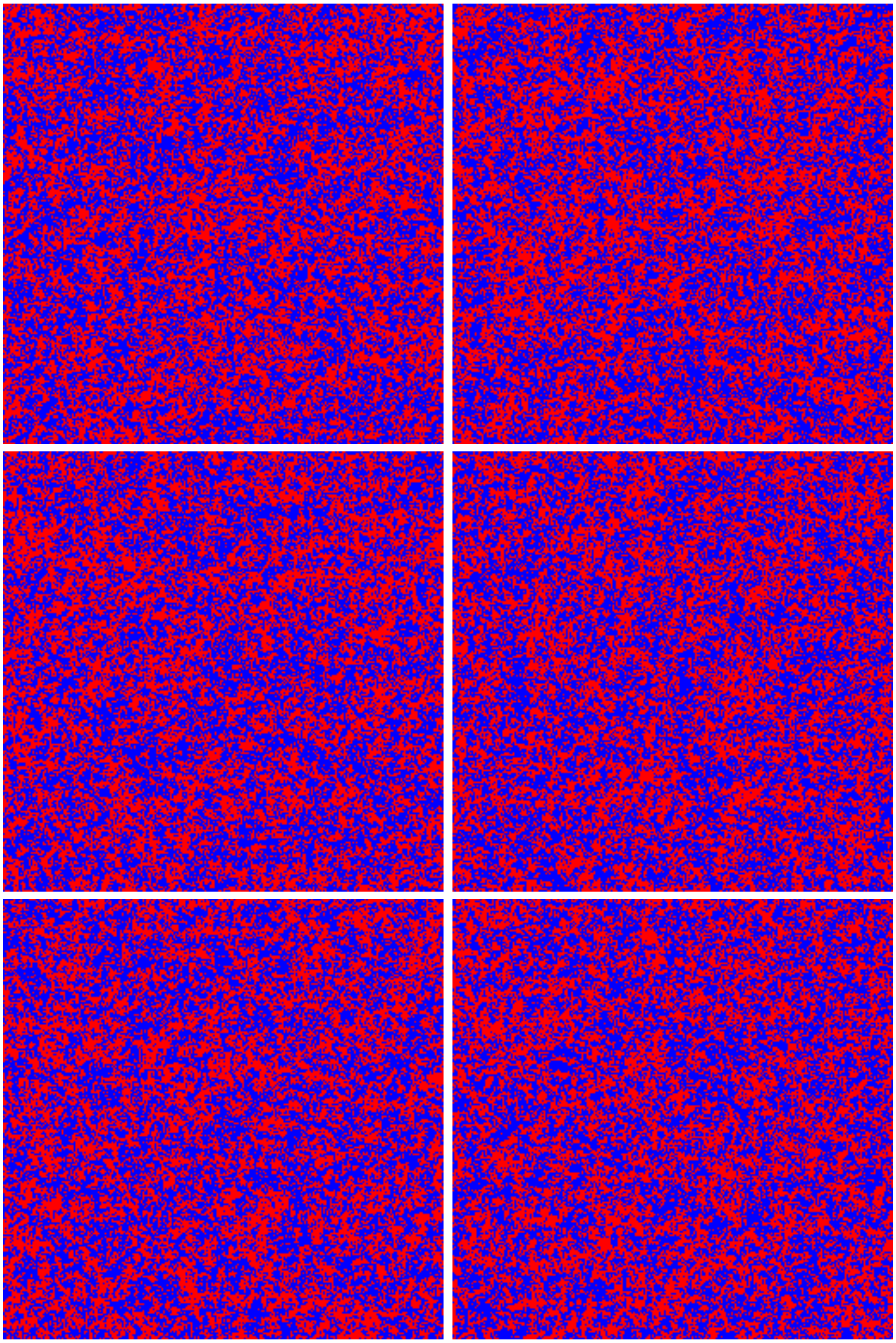

Figure S7. 256×256 windows of the surface of an $840 \times 840 \times 840$ 3D clean Ising model at $T = T_c$

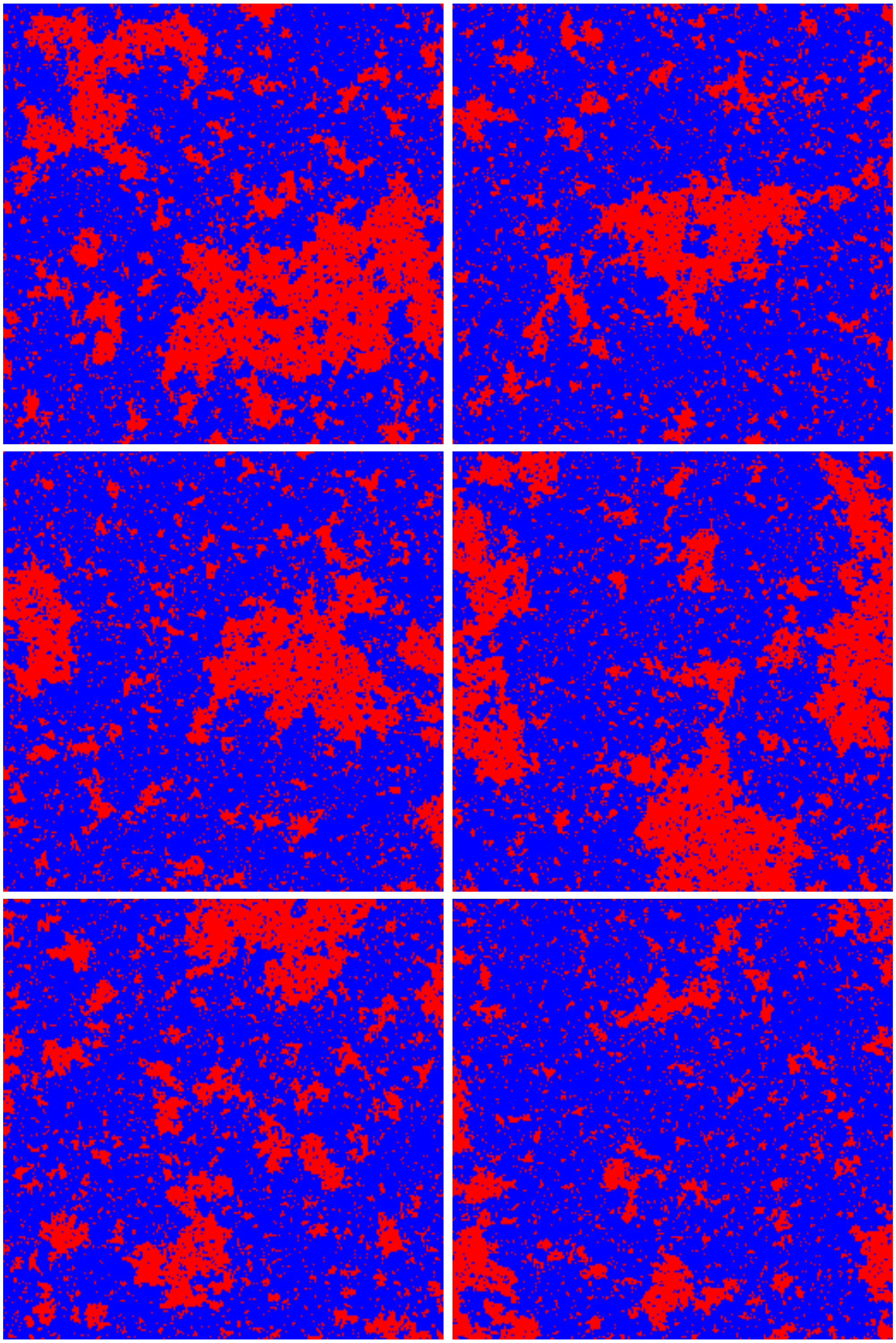

Figure S8. 256×256 windows of a 1000×1000 2D clean Ising model at $T = T_c$

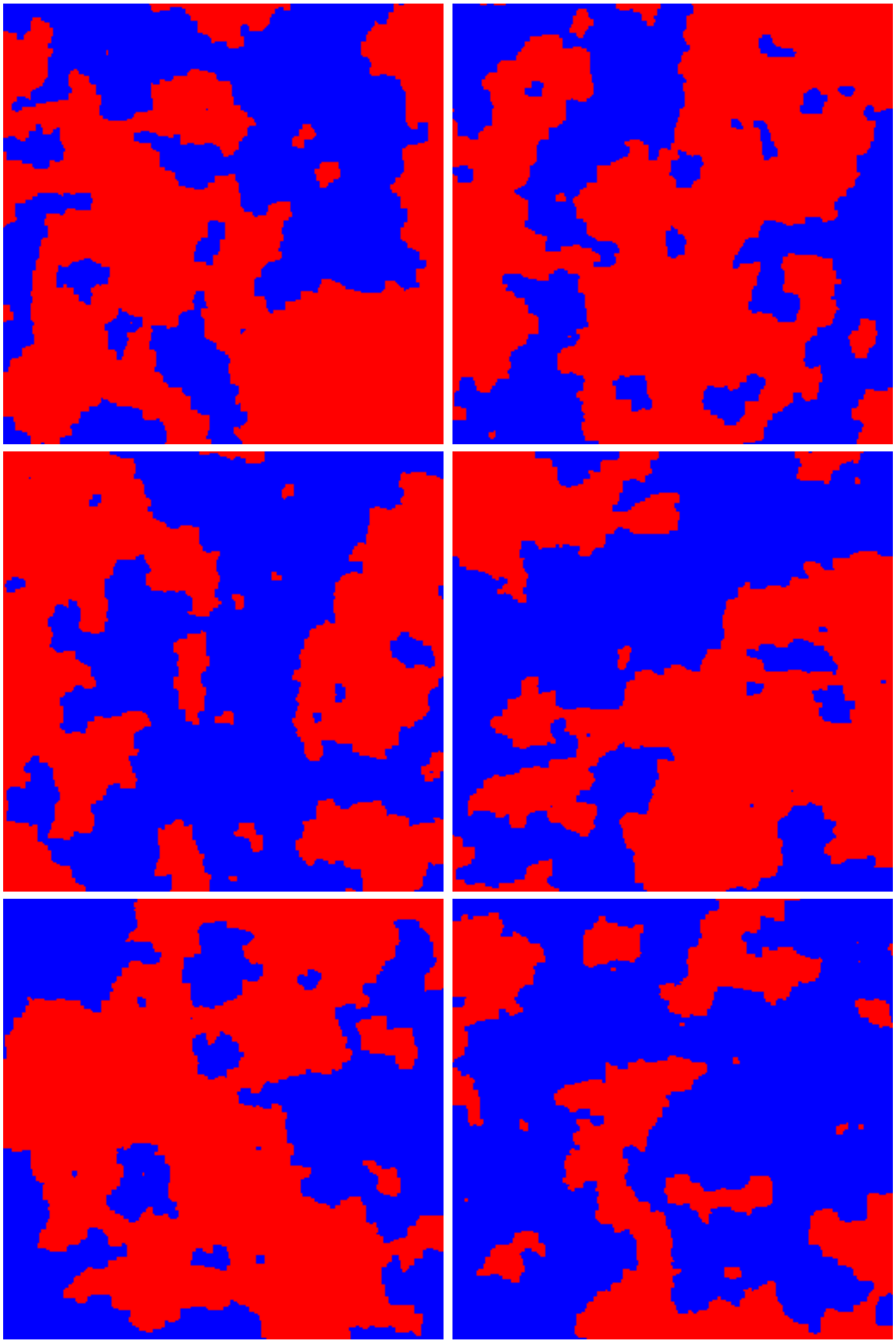

Figure S9. 256 $\times$ 256 windows of a 512 $\times$ 512 2D random field Ising model at $T = 0$ and $R = 1$. Simulations run at $R = 0.5$ and $R = 0.6$ produced completely magnetized configurations for this size system.

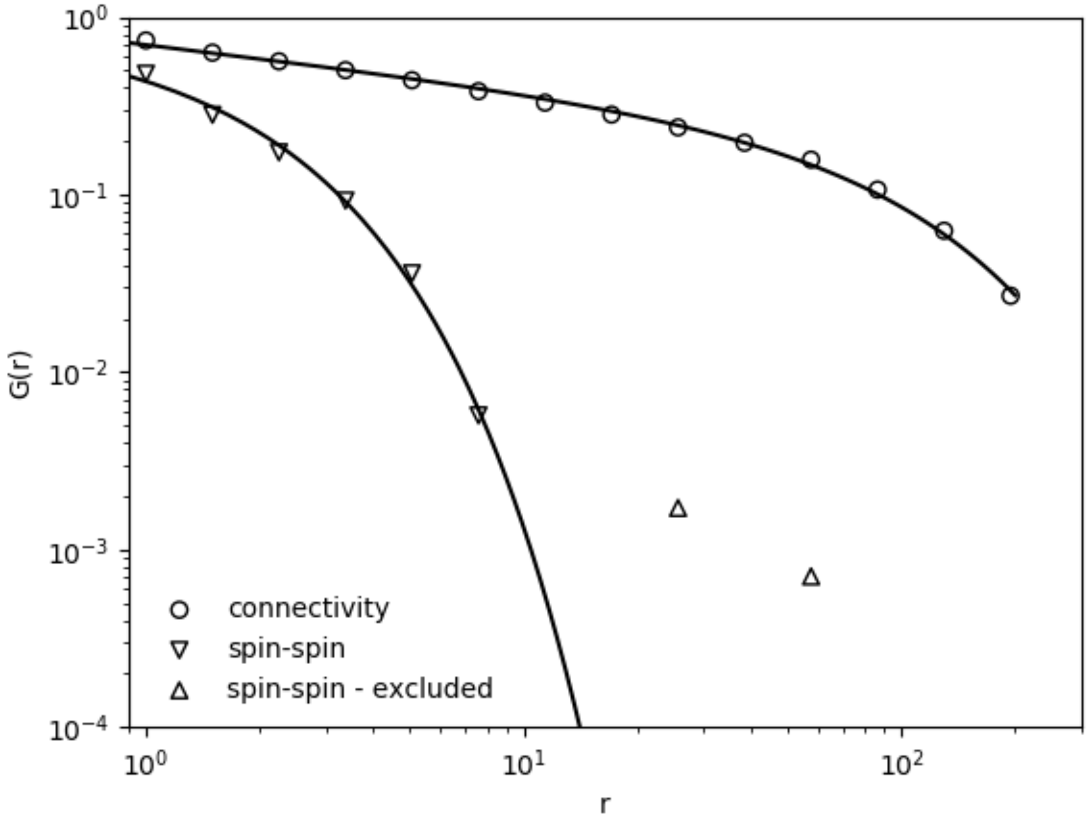

Figure S10. An example of the pair connectivity function and the spin-spin correlation function for a 256×256 window on a free surface of a $512 \times 512 \times 512$ 3D RFIM system with open boundary conditions on the top and bottom surface and periodic boundary conditions in all other directions. Here, $T = 0$ and the disorder strength $\Delta = 3J$ (see Eqn. 2 in the main text). The open circles represent the pair connectivity function $G_{\text{conn}}(r)$, and the open triangles represent the spin-spin correlation function, $G_{\text{spin}}(r)$. The solid lines are fits to a power law times an exponential, $G(r) \propto e^{-r/\xi}/r^{d-2+\eta}$. Points beyond $r = 11$ sites have been excluded from the fit of the spin-spin correlation function, because the value has effectively become zero beyond that point, with many values being negative, but all bounded by $|G_{\text{spin}}(r)| \lesssim .002$. Note that while the spin-spin correlation function is not robustly power law, the pair connectivity function is. Here, $\xi_{\text{spin}} = 1.59 \pm 0.22$ while $\xi_{\text{pair}} = 103.1 \pm 4.3$.

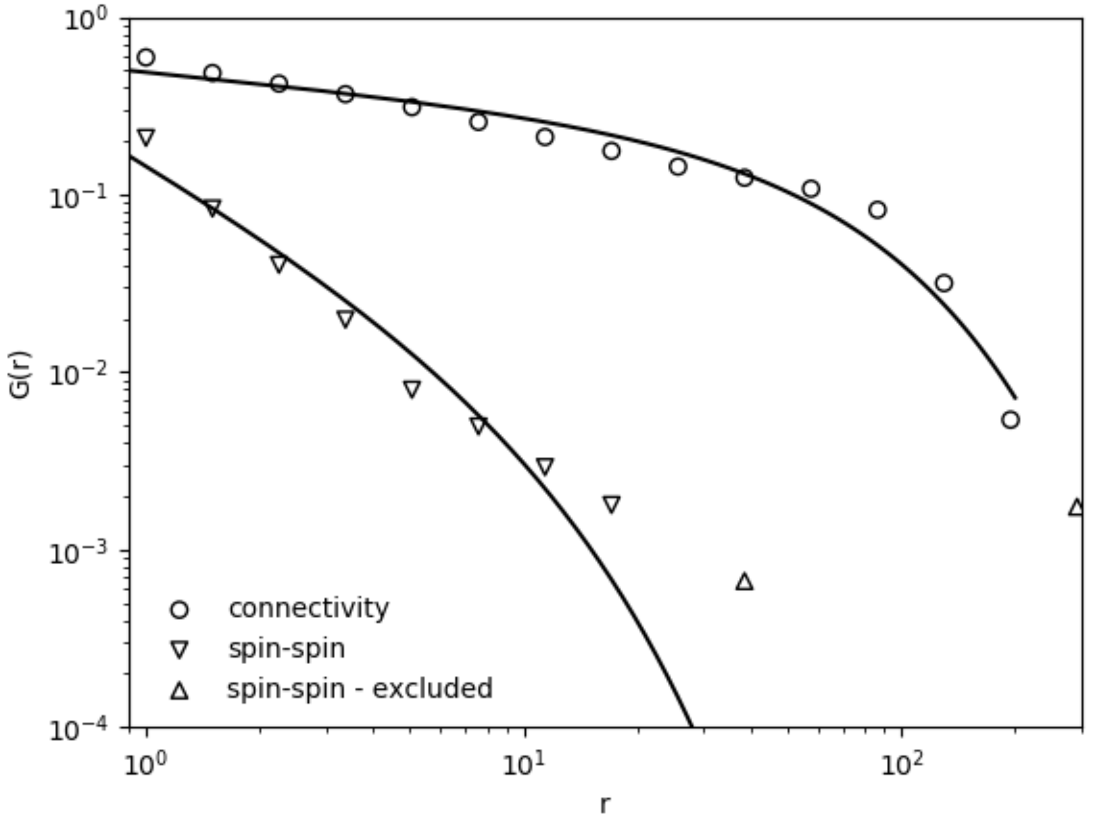

Figure S11. An example of the pair connectivity function and the spin-spin correlation function for a 256×256 window on a free surface of a $840 \times 840 \times 840$ 3D clean Ising model with open boundary conditions on the top and bottom surface and periodic boundary conditions in all other directions. Here, $T = 4.51J \lesssim T_c = 4.51152786J$ [66, 67]. The open circles represent the pair connectivity function $G_{\rm conn}(r)$, and the open triangles represent the spin-spin correlation function, $G_{\rm spin}(r)$. The solid lines are fits to a power law times an exponential, $G(r) \propto e^{-r/\xi}/r^{d-2+\eta}$. Points beyond $r = 25$ sites have been excluded from the fit of the spin-spin correlation function, because the value has effectively become zero beyond that point, with many values being negative, but all bounded by $|G_{\rm spin}(r)| \lesssim 0.0005$. Note that while the spin-spin correlation function is not robustly power law, the pair connectivity function is. Here, $\xi_{\rm spin} = 8.2 \pm 3.9$ while $\xi_{\rm pair} = 62.7 \pm 8.3$. The fact that the spin-spin correlation function shows less than a decade of scaling even within 0.03% of T_c is indicative of how narrow the critical region is for C-3D.

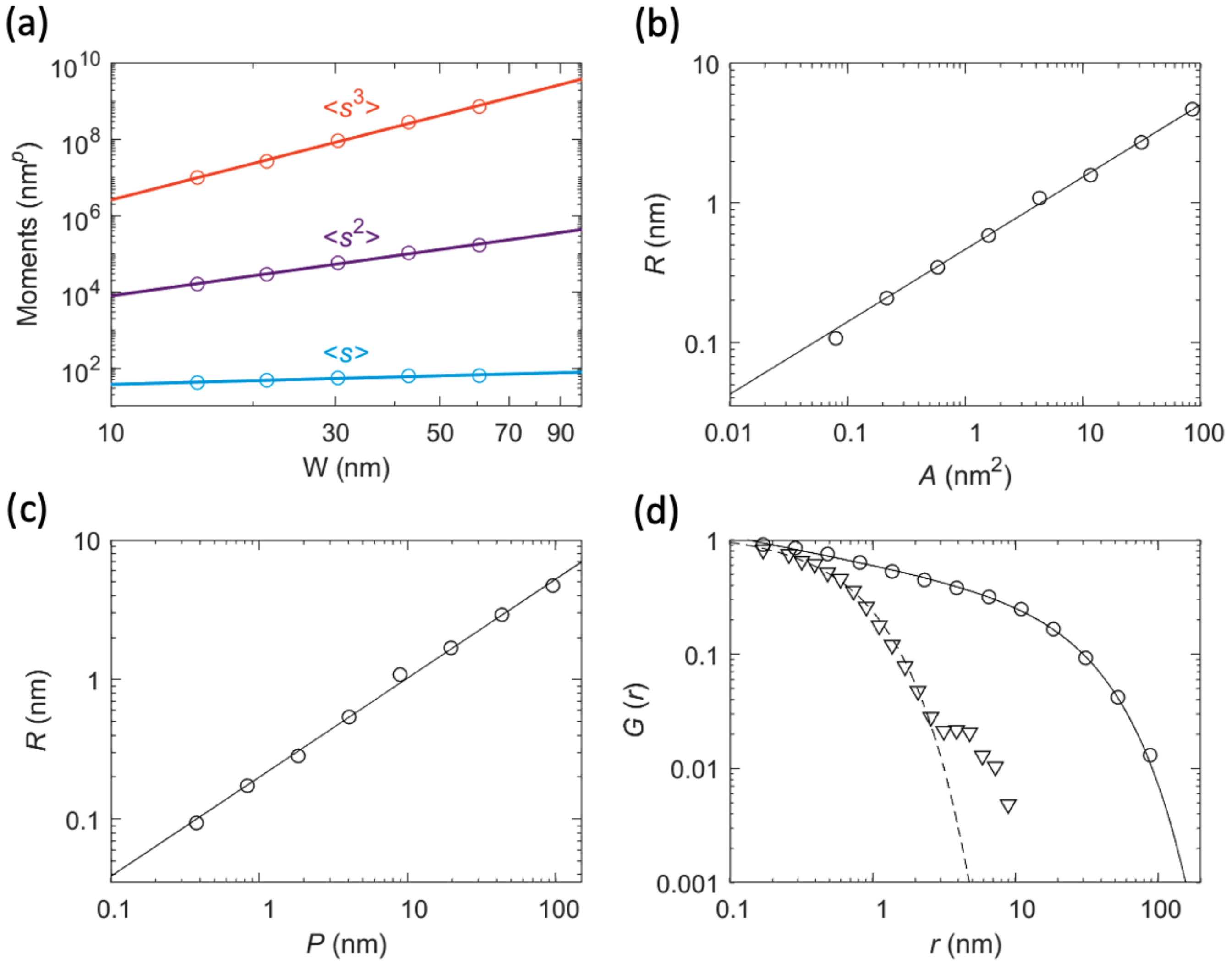

Figure S12. **Cluster structure and power-law statistical analysis in UD25K sample.** Data are from the R-maps in Fig. S2(a). (**a**) Finite-size-scaling of moments for cluster size distribution, from which the Fisher exponent τ is calculated. Here p corresponds to the power indexes for the first ($p = 1$), second ($p = 2$) and third ($p = 3$) moments. (**b**) The radius of gyration R versus cluster area A showing a power law between them, from which the critical exponent d_v^* is extracted. (**c**) The radius of gyration R versus effective cluster perimeter P. The perimeter P also shows a power law dependence on R, from which the critical exponent d_h^* is extracted. (**d**) Spatial correlation functions $G_{\text{conn}}(\mathbf{r})$ (circles) and $G_{\text{spin}}(\mathbf{r})$ (triangles) for calculating the critical exponent $d - 2 + \eta_{||}$. The black line shows the best fit of the pair connectivity correlation function by $G_{\text{conn}} \propto r^{-(d-2+\eta_{||})}\exp(-r/\xi)$, whereas the dashed line is only a guide to the eye. Logarithmic binning has been used in (**b-d**) [48].

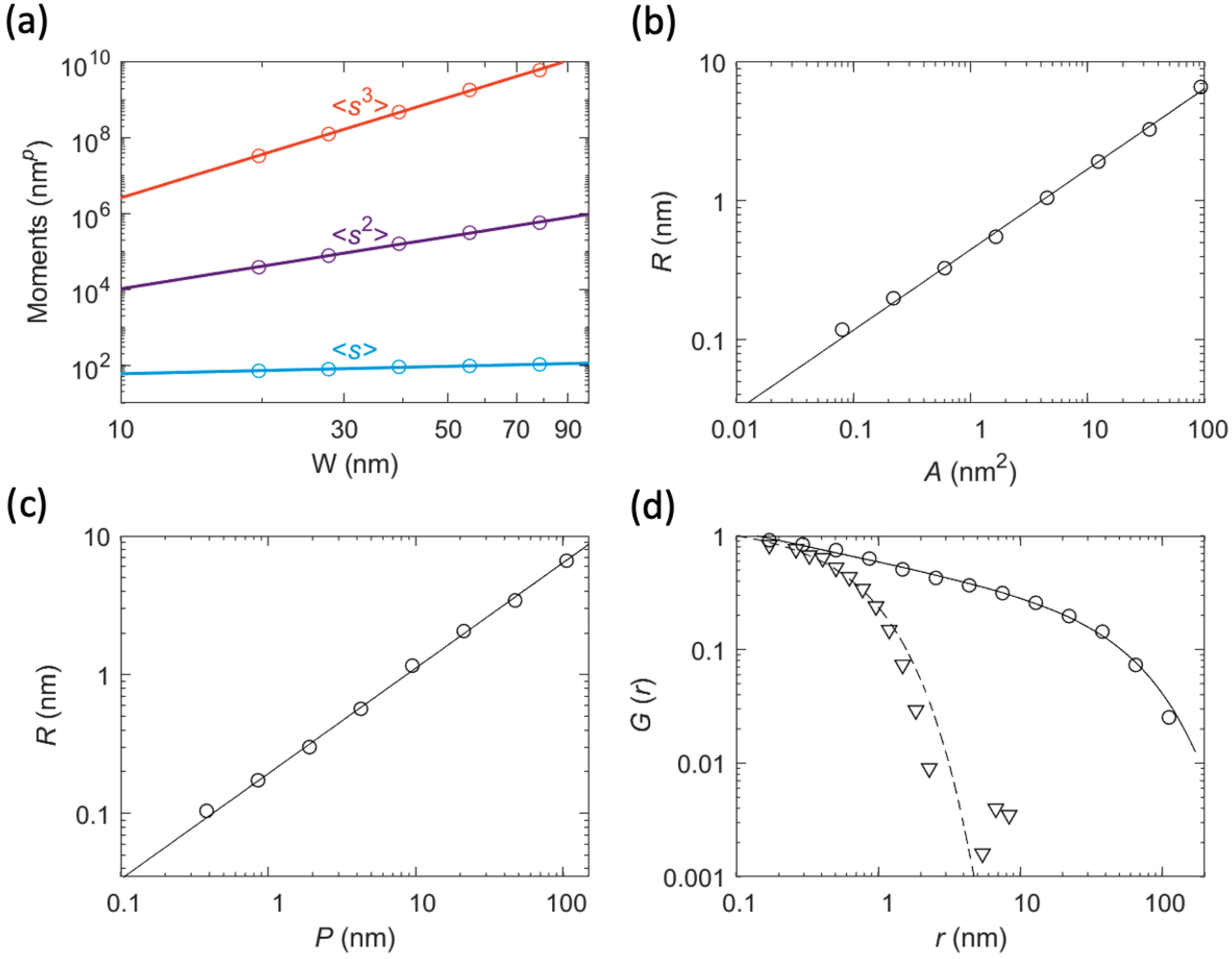

Figure S13. **Cluster structure and power-law statistical analysis in OPT35K sample.** Data are from the R-maps in Fig. S2(c). (**a**) Finite-size-scaling of moments for cluster size distribution, from which the Fisher exponent τ is calculated. Here p corresponds to the power indexes for the first ($p = 1$), second ($p = 2$) and third ($p = 3$) moments. (**b**) The radius of gyration R versus cluster area A showing a power law between them, from which the critical exponent d_v^* is extracted. (**c**) The radius of gyration R versus effective cluster perimeter P. The perimeter P also shows a power law dependence on R, from which the critical exponent d_h^* is extracted. (**d**) Spatial correlation functions $G_{\text{conn}}(\mathbf{r})$ (circles) and $G_{\text{spin}}(\mathbf{r})$ (triangles) for calculating the critical exponent $d - 2 + \eta_{||}$. The black line shows the best fit of the pair connectivity correlation function by $G_{\text{conn}} \propto r^{-(d-2+\eta_{||})}\exp(-r/\xi)$, whereas the dashed line is only a guide to the eye. Logarithmic binning has been used in (**b-d**) [48].

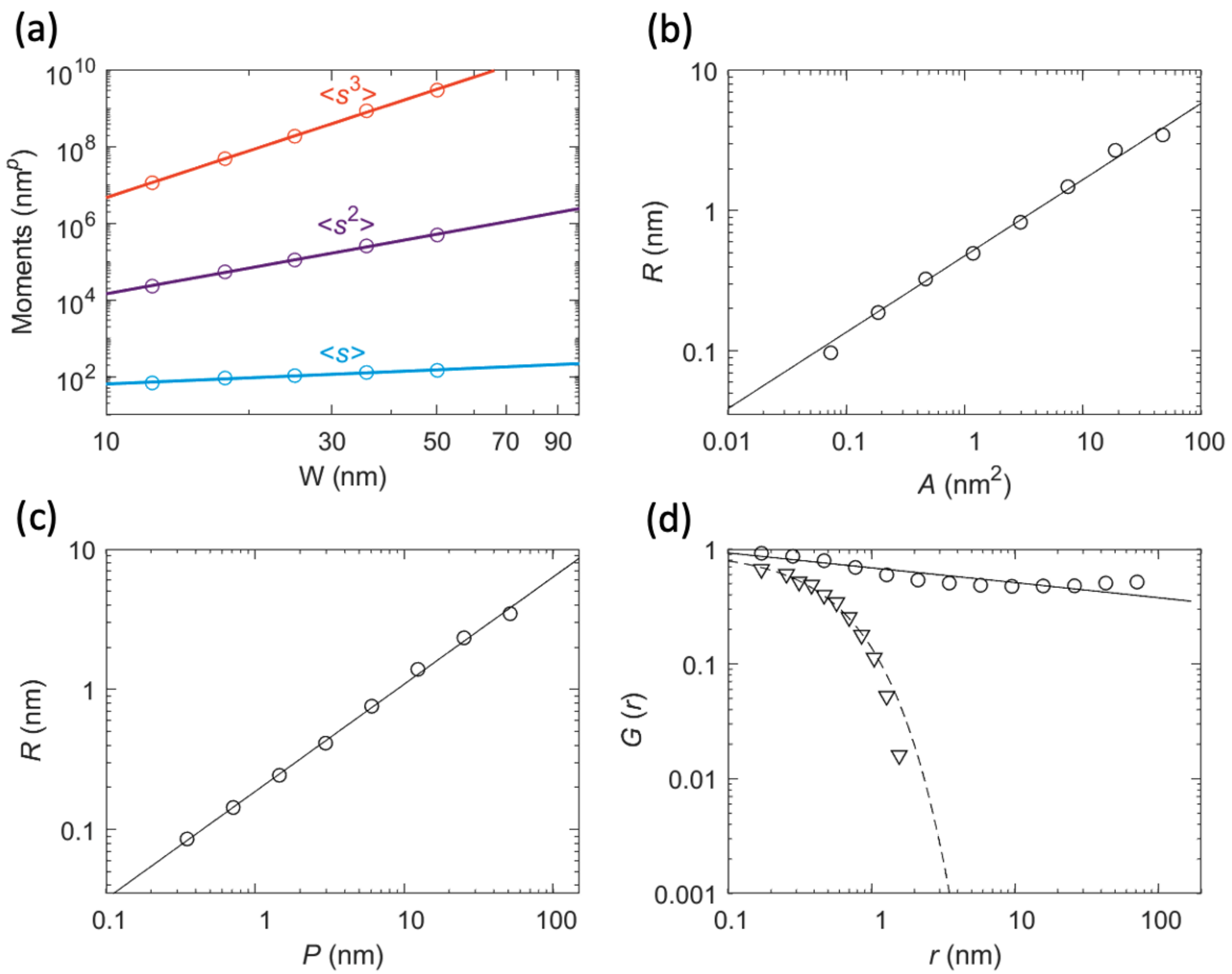

Figure S14. **Cluster structure and power-law statistical analysis in OD16K sample.** Data are from the R-maps in Fig. S2(d). (**a**) Finite-size-scaling of moments for cluster size distribution, from which the Fisher exponent τ is calculated. Here p corresponds to the power indexes for the first ($p = 1$), second ($p = 2$) and third ($p = 3$) moments. (**b**) The radius of gyration R versus cluster area A showing a power law between them, from which the critical exponent d_v^* is extracted. (**c**) The radius of gyration R versus effective cluster perimeter P. The perimeter P also shows a power law dependence on R, from which the critical exponent d_h^* is extracted. (**d**) Spatial correlation functions $G_{\rm conn}(\mathbf{r})$ (circles) and $G_{\rm spin}(\mathbf{r})$ (triangles) for calculating the critical exponent $d - 2 + \eta_{||}$. The black line shows the best fit of the pair connectivity correlation function by $G_{\rm conn} \propto r^{-(d-2+\eta_{||})}\exp(-r/\xi)$, whereas the dashed line is only a guide to the eye. Logarithmic binning has been used in (**b-d**) [48].

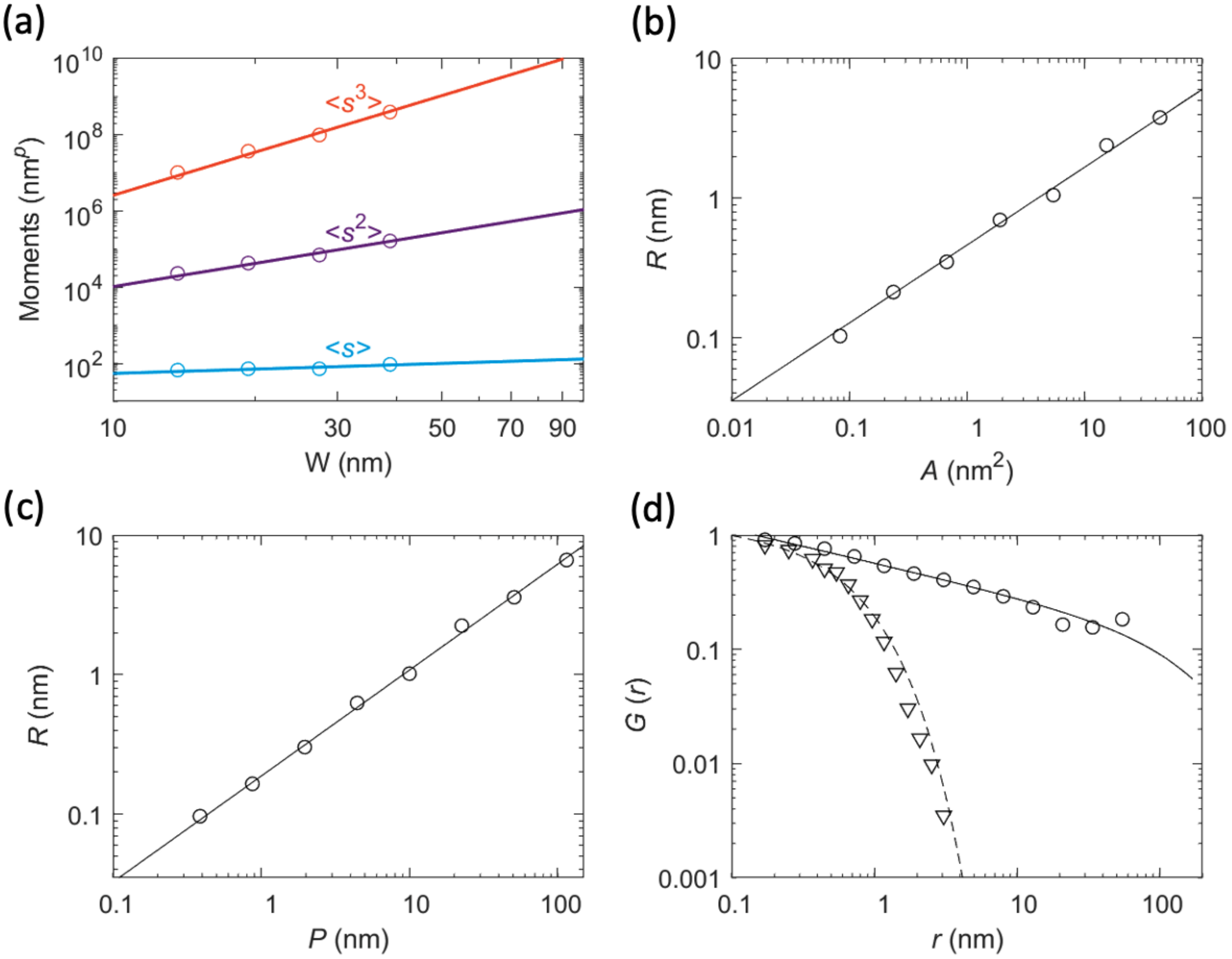

Figure S15. **Cluster structure and power-law statistical analysis in OD15K sample.** Data are from the R-maps in Fig. S2(e). (**a**) Finite-size-scaling of moments for cluster size distribution, from which the Fisher exponent τ is calculated. Here p corresponds to the power indexes for the first ($p = 1$), second ($p = 2$) and third ($p = 3$) moments. (**b**) The radius of gyration R versus cluster area A showing a power law between them, from which the critical exponent d_v^* is extracted. (**c**) The radius of gyration R versus effective cluster perimeter P. The perimeter P also shows a power law dependence on R, from which the critical exponent d_h^* is extracted. (**d**) Spatial correlation functions $G_{\text{conn}}(\mathbf{r})$ (circles) and $G_{\text{spin}}(\mathbf{r})$ (triangles) for calculating the critical exponent $d - 2 + \eta_{||}$. The black line shows the best fit of the pair connectivity correlation function by $G_{\text{conn}} \propto r^{-(d-2+\eta_{||})}\exp(-r/\xi)$, whereas the dashed line is only a guide to the eye. Logarithmic binning has been used in (**b-d**) [48].

SUPPLEMENTARY REFERENCES

[1] J. E. Hoffman, *et al.* A four unit cell periodic pattern of quasi-particle states surrounding vortex cores in $Bi_2Sr_2CaCu_2O_{8+d}$. Science **295**, 466 (2002).

[2] C. Howald, H. Eisaki, N. Kaneko, and A. Kapitulnik. Coexistence of periodic modulation of quasiparticle states and superconductivity in $Bi_2Sr_2CaCu_2O_{8+d}$. Proceedings of the National Academy of Sciences **100**, 9705 (2003).

[3] Y. Kohsaka, *et al.* An intrinsic bond-centered electronic glass with unidirectional domains in underdoped cuprates. Science **315**, 1380 (2007).

[4] W. D. Wise, *et al.* Charge-density-wave origin of cuprate checkerboard visualized by scanning tunnelling microscopy. Nature Physics **4**, 696 (2008).

[5] T. Hanaguri, *et al.* A checkerboard electronic crystal state in lightly hole-doped $Ca_{2-x}Na_xCuO_2Cl_2$. Nature **430**, 1001 (2004).

[6] T. Wu, *et al.* Magnetic-field-induced charge-stripe order in the high-temperature superconductor $YBa_2Cu_3O_y$. Nature **477**, 191 (2011).

[7] J. Chang, *et al.* Direct observation of competition between superconductivity and charge density wave order in $YBa_2Cu_3O_{6.67}$. Nature Physics **8**, 871 (2012).

[8] G. Ghiringhelli, *et al.* Long-Range Incommensurate Charge Fluctuations in (Y,Nd)$Ba_2Cu_3O_{6+x}$. Science **337**, 821 (2012).

[9] R. Comin, *et al.* Charge Order Driven by Fermi-Arc Instability in $Bi_2Sr_{2-x}La_xCuO_{6+\delta}$. Science **343**, 390 (2014).

[10] E. H. da Silva Neto, *et al.* Charge ordering in the electron-doped superconductor $Nd_{2-x}Ce_xCuO_4$. Science **347**, 282 (2015).

[11] T. P. Croft, C. Lester, M. S. Senn, A. Bombardi, and S. M. Hayden. Charge density wave fluctuations in $La_{2-x}Sr_xCuO_4$ and their competition with superconductivity. Physical Review B **89**, 224513 (2014).

[12] W. Tabis, *et al.* Charge order and its connection with Fermi-liquid charge transport in a pristine high-T_c cuprate. Nature Communications **5**, 5875 (2014).

[13] Y. Y. Peng, *et al.* Direct observation of charge order in underdoped and optimally doped $Bi_2(Sr,La)_2CuO_{6+\delta}$ by resonant inelastic x-ray scattering. Physical Review B **94**, 184511 (2016).

[14] M. Kang, *et al.* Evolution of charge order topology across a magnetic phase transition in cuprate superconductors. Nature Physics **15**, 335 (2019).

[15] J. Li, *et al.* Multiorbital charge-density wave excitations and concomitant phonon anomalies in $Bi_2Sr_2LaCuO_{6+\delta}$. Proceedings of the National Academy of Sciences **117**, 16219 (2020).

[16] P. Abbamonte, *et al.* Spatially modulated 'Mottness' in $La_{2-x}Ba_xCuO_4$. Nature physics **1**, 155 (2005).

[17] R. Comin, *et al.* Symmetry of charge order in cuprates. Nature materials **14**, 796 (2015).

[18] E. H. da Silva Neto, *et al.* Ubiquitous Interplay Between Charge Ordering and High-Temperature Superconductivity in Cuprates. Science **343**, 393 (2014).

[19] B. Phillabaum, E. W. Carlson, and K. A. Dahmen. Spatial complexity due to bulk electronic nematicity in a superconducting underdoped cuprate. Nature Communications **3**, 915 (2012).

[20] M. J. Lawler, *et al.* Intra-unit-cell electronic nematicity of the high-T_c copper-oxide pseudogap states. Nature **466**, 347 (2010).

[21] C. V. Parker, *et al.* Fluctuating stripes at the onset of the pseudogap in the high-T_c superconductor $Bi_2Sr_2CaCu_2O_{8+d}$. Nature **468**, 677 (2010).

[22] K. Fujita, *et al.* Direct phase-sensitive identification of a d-form factor density wave in underdoped cuprates. Proceedings of the National Academy of Sciences **111**, E3026 (2014).

[23] J. M. Tranquada, B. J. Sternlieb, J. D. Axe, Y. Nakamura, and S. Uchida. Evidence for stripe correlations of spins and holes in copper oxide superconductors. Nature **375**, 561 (1995).

[24] H. A. Mook, P. Dai, and F. Dog. Spin fluctuations in $YBa_2Cu_3O_{6.6}$. Nature **395**, 580 (1998).

[25] R. Comin, *et al.* Broken translational and rotational symmetry via charge stripe order in underdoped $YBa_2Cu_3O_{6+y}$. Science **347**, 1335 (2015).

[26] C. Howald, H. Eisaki, N. Kaneko, M. Greven, and A. Kapitulnik. Periodic density-of-states modulations in superconducting $Bi_2Sr_2CaCu_2O_{8+\delta}$. Phys. Rev. B **67**, 014533 (2003).

[27] M. Vershinin, *et al.* Local Ordering in the Pseudogap State of the High-T_c Superconductor $Bi_2Sr_2CaCu_2O_{8+\delta}$. Science **303**, 1995 (2004).

[28] R. Arpaia, *et al.* Dynamical charge density fluctuations pervading the phase diagram of a Cu-based high-T_c superconductor. Science **365**, 906 (2019).

[29] S. Gerber, *et al.* Three-dimensional charge density wave order in $YBa_2Cu_3O_{6.67}$ at high magnetic fields. Science **350**, 949 (2015).

[30] H. Jang, *et al.* Ideal charge-density-wave order in the high-field state of superconducting YBCO. Proceedings of the National Academy of Sciences **113**, 14645 (2016).

[31] J.-J. Wen, *et al.* Observation of two types of charge-density-wave orders in superconducting $La_{2-x}Sr_xCuO_4$. Nature Communications **10**, 3269 (2019).

[32] J. Robertson, S. Kivelson, E. Fradkin, A. Fang, and A. Kapitulnik. Distinguishing patterns of charge order: Stripes or

checkerboards. Physical Review B **74**, 134507 (2006).
[33] A. Del Maestro, B. Rosenow, and S. Sachdev. From stripe to checkerboard ordering of charge-density waves on the square lattice in the presence of quenched disorder. Physical Review B **74**, 024520 (2006).
[34] M. E. Fisher. The theory of condensation and the critical point. Physics Physique Fizika **3**, 255 (1967).
[35] D. Stauffer and A. Aharony. *Introduction to percolation theory.* CRC Press (1994).
[36] S. Liu, E. W. Carlson, and K. A. Dahmen. Connecting Complex Electronic Pattern Formation to Critical Exponents. Condensed Matter **6**, 39 (2021).
[37] E. W. Carlson, V. J. Emery, S. A. Kivelson, and D. Orgad. *Concepts in High Temperature Superconductivity.* Springer-Verlag (2004).
[38] V. J. Emery, S. A. Kivelson, and O. Zachar. Spin-gap proximity effect mechanism of high-temperature superconductivity. Physical Review B **56**, 6120 (1997).
[39] S. A. Kivelson, E. Fradkin, and V. J. Emery. Electronic liquid-crystal phases of a doped Mott insulator. Nature **393**, 550 (1998).
[40] O. Perković, K. Dahmen, and J. Sethna. Avalanches, Barkhausen Noise, and Plain Old Criticality. Physical Review Letters **75**, 4528 (1995).
[41] L. Nie, G. Tarjus, and S. A. Kivelson. Quenched disorder and vestigial nematicity in the pseudogap regime of the cuprates. Proceedings of the National Academy of Sciences **111**, 7980 (2014).
[42] W. S. Lee, *et al.* Spectroscopic fingerprint of charge order melting driven by quantum fluctuations in a cuprate. Nature Physics **17**, 53 (2021).
[43] E. Carlson, K. A. Dahmen, E. Fradkin, and S. Kivelson. Hysteresis and Noise from Electronic Nematicity in High-Temperature Superconductors. Physical Review Letters **96**, 097003 (2006).
[44] E. W. Carlson, S. Liu, B. Phillabaum, and K. A. Dahmen. Decoding Spatial Complexity in Strongly Correlated Electronic Systems. Journal of Superconductivity and Novel Magnetism **28**, 1237–1243 (2015).
[45] P. Elias, A. Feinstein, and C. Shannon. A note on the maximum flow through a network. IRE Transactions on Information Theory **2**, 117 (1956).
[46] A. V. Goldberg. Two-Level Push-Relabel Algorithm for the Maximum Flow Problem. In *Algorithmic Aspects in Information and Management*, Lecture Notes in Computer Science, 212–225. Springer-Verlag, Berlin, Heidelberg (2009).
[47] J. C. Picard and H. D. Ratliff. Minimum cuts and related problems. Networks **5**, 357 (1975).
[48] M. E. J. Newman. Power laws, Pareto distributions and Zipf's law. Contemporary Physics **46**, 323 (2005).
[49] T. Hacker, B. Yang, and G. McCartney. Empowering Faculty: A Campus Cyberinfrastructure Strategy for Research Communities. Educause Review (2014).
[50] M. Presland, J. Tallon, R. Buckley, R. Liu, and N. Flower. General trends in oxygen stoichiometry effects on T_c in Bi and Tl superconductors. Physica C: Superconductivity **176**, 95 (1991).
[51] Y. Kohsaka, *et al.* How Cooper pairs vanish approaching the Mott insulator in $Bi_2Sr_2CaCu_2O_{8+d}$. Nature **454**, 1072 (2008).
[52] Y. He, *et al.* Fermi surface and pseudogap evolution in a cuprate superconductor. Science **344**, 608 (2014).
[53] T. Hanaguri, *et al.* Quasiparticle interference and superconducting gap in $Ca_{2-x}Na_xCuO_2Cl_2$. Nature Physics **3**, 865 (2007).
[54] J. Cardy. *Scaling and Renormalization in Statistical Physics.* Cambridge University Press, Cambridge (1996).
[55] W. Janke and A. M. J. Schakel. Fractal structure of spin clusters and domain walls in the two-dimensional Ising model. Physical Review E **71**, 385 (2005).
[56] D. Stauffer and A. Aharony. *Introduction To Percolation Theory.* Taylor & Francis (2018).
[57] D. Stauffer. Scaling theory of percolation clusters. Journal of Magnetism and Magnetic Materials .
[58] T. Grossman and A. Aharony. Accessible external perimeters of percolation clusters. Journal of Physics A: Mathematical and General **20**, L1193 (1999).
[59] L. Környei and F. Iglói. Geometrical clusters in two-dimensional random-field Ising models. Physical Review E **75**, 25 (2007).
[60] H. Ji and M. O. Robbins. Transition from compact to self-similar growth in disordered systems: Fluid invasion and magnetic-domain growth. Physical Review A **44**, 2538 (1991).
[61] E. T. Seppälä, M. J. Alava, and E. T. Seppala. Susceptibility and percolation in two-dimensional random field Ising magnets. Physical Review E **63**, 066109 (2001).
[62] B. Drossel and K. Dahmen. Depinning of a domain wall in the 2d random-field Ising model. The European Physical Journal B - Condensed Matter and Complex Systems **3**, 485 (1998).
[63] D. Stauffer. Scaling theory of percolation clusters. Physics Reports **54**, 1 (1979).
[64] W. Janke and A. M. J. Schakel. Fractal structure of spin clusters and domain walls in the two-dimensional Ising model. Physical Review E **71**, 036703 (2005).
[65] A. A. Saberi and H. Dashti-Naserabadi. Three Dimensional Ising Model, Percolation Theory and Conformal Invariance. Europhysics Letters **92**, 67005 (2010).
[66] F. Livet. The Cluster Updating Monte Carlo Algorithm Applied to the 3d Ising Problem. Europhysics Letters **16**, 139 (1991).
[67] A. L. Talapov and H. W. J. Blöte. The magnetization of the 3D Ising model. Journal of Physics A: Mathematical and General **29**, 5727 (1996).
